# Adverse Health-Related Quality of Life Outcome Despite Adequate Clinical Response to Treatment in Systemic Lupus Erythematosus

**DOI:** 10.3389/fmed.2021.651249

**Published:** 2021-04-16

**Authors:** Alvaro Gomez, Victor Qiu, Arvid Cederlund, Alexander Borg, Julius Lindblom, Sharzad Emamikia, Yvonne Enman, Jon Lampa, Ioannis Parodis

**Affiliations:** ^1^Division of Rheumatology, Department of Medicine Solna, Karolinska Institutet, Stockholm, Sweden; ^2^Department of Gastroenterology, Dermatology and Rheumatology, Karolinska University Hospital, Stockholm, Sweden

**Keywords:** systemic lupus erythematosus, health-related quality of life, patient-reported outcome, fatigue, biologic drugs, patient perscpective

## Abstract

**Objective:** To determine the prevalence of adverse health-related quality of life (HRQoL) outcomes in patients with SLE who achieved an adequate clinical response after a 52-week long standard therapy plus belimumab or placebo, and identify contributing factors.

**Methods:** We included patients who met the primary endpoint of the BLISS-52 (NCT00424476) and BLISS-76 (NCT00410384) trials, i.e., SLE Responder Index 4 (total population: *N* = 760/1,684; placebo: *N* = 217/562; belimumab 1 mg/kg: *N* = 258/559; belimumab 10 mg/kg: *N* = 285/563). Adverse HRQoL outcomes were defined as SF-36 scale scores ≤ the 5th percentile derived from age- and sex-matched population-based norms, and FACIT-Fatigue scores <30. We investigated factors associated with adverse HRQoL outcomes using logistic regression analysis.

**Results:** We found clinically important diminutions of HRQoL in SLE patients compared with matched norms and high frequencies of adverse HRQoL outcomes, the highest in SF-36 general health (29.1%), followed by FACIT-Fatigue (25.8%) and SF-36 physical functioning (25.4%). Overall, frequencies were higher with increasing age. Black/African American and White/Caucasian patients reported higher frequencies than Asians and Indigenous Americans, while Hispanics experienced adverse HRQoL outcome less frequently than non-Hispanics. Established organ damage was associated with adverse physical but not mental HRQoL outcomes; particularly, damage in the cardiovascular (OR: 2.12; 95% CI: 1.07–4.21; *P* = 0.032) and musculoskeletal (OR: 1.41; 95% CI: 1.01–1.96; *P* = 0.041) domains was associated with adverse SF-36 physical component summary. Disease activity showed no impact on HRQoL outcomes. In multivariable logistic regression analysis, addition of belimumab to standard therapy was associated with lower frequencies of adverse SF-36 physical functioning (OR: 0.59; 95% CI: 0.39–0.91; *P* = 0.016) and FACIT-F (OR: 0.53; 95% CI: 0.34–0.81; *P* = 0.004).

**Conclusions:** Despite adequate clinical response to standard therapy plus belimumab or placebo, a substantial proportion of SLE patients still reported adverse HRQoL outcomes. While no impact was documented for disease activity, established organ damage contributed to adverse outcome within physical HRQoL aspects and add-on belimumab was shown to be protective against adverse physical functioning and severe fatigue.

## Introduction

Systemic lupus erythematosus (SLE) is a chronic autoimmune disease with a highly heterogeneous clinical presentation. A better understanding of the subtleties of the disease together with improvements in medical care have contributed to prolonged life expectancy for SLE patients over the past decades (1). However, people living with SLE still suffer from substantial diminutions of health-related quality of life (HRQoL) compared with the general population and with other chronic diseases (2).

Divergent disease prevalence, clinical manifestations, disease course and mortality rates between sexes and across ethnic groups contribute to the heterogeneity of SLE (3, 4). Overall, patients of Black/African American or Asian origin have an increased risk for SLE than White/Caucasian individuals, and show more severe disease phenotypes. The implications of these discrepancies across ethnic groups in SLE patients' HRQoL perception have not been thoroughly delineated.

Some studies have demonstrated that conventional synthetic and biological disease-modifying agents contribute to improvements in SLE patients' HRQoL (5–9), and responders to treatment have been shown to report greater improvements than non-responders (10, 11). Although these observations are clinically relevant, improvement following a therapeutic intervention does not necessarily signify that the individual has achieved a satisfactory health perception. In rheumatoid arthritis (RA), significant pain and severe fatigue persist in a substantial proportion of patients who achieve a good clinical response to treatment or remission (12, 13). This paradoxical observation has not been thoroughly explored in SLE.

In the present investigation, we aimed to determine the prevalence of adverse HRQoL outcomes in patients with SLE who achieved an adequate clinical response after a 52-week long period on standard therapy (ST) plus belimumab or placebo within the frame of two phase III clinical trials. We further compared frequencies of adverse HRQoL outcomes across different age categories and ethnic groups, and sought to identify contributing factors.

## Materials and Methods

### Study Design and Population

We designed a *post-hoc* analysis of data from two randomised, double blind, phase III clinical trials, i.e., BLISS-52 (NCT00424476) (14) and BLISS-76 (NCT00410384) (15), which comprised 865 and 819 SLE patients, respectively. Inclusion criteria for both trials were age ≥18 years, SLE diagnosis according to the revised 1997 American College of Rheumatology (ACR) criteria (16), an active disease defined as a Safety of Estrogens in Lupus National Assessment Systemic Lupus Erythematosus Disease Activity Index (SELENA-SLEDAI) (17) score ≥6, and antinuclear antibody (ANA) titre ≥1:80 and/or serum anti-double stranded (ds)DNA antibody level ≥30 IU/mL. Key exclusion criteria included pregnancy, severe active lupus nephritis and active neuropsychiatric SLE. Patients mainly from Asia, Eastern Europe and Latin America were enrolled in BLISS-52, and from North America and Europe in BLISS-76.

The primary endpoint of the trials was achievement of the SLE responder index 4 (SRI-4) at week 52, defined as ≥4 points reduction in the SELENA-SLEDAI score compared with baseline, no new classic British Isles Lupus Assessment Group Index (BILAG) (18) A organ domain score and no more than one new BILAG B organ domain scores compared with baseline, and no worsening in the physician's global assessment (PGA) by ≥0.30 points (range: 0–3) from baseline. Of 1,684 study participants, 760 met the criteria for SRI-4 response at week 52, and constituted the study population of this *post-hoc* analysis. The frequency of SRI-4 responders was 217/562 in the placebo arm, 258/559 in the belimumab 1 mg/kg arm, and 285/563 in the belimumab 10 mg/kg arm. Accordingly, evaluation of adverse HRQoL outcomes was based on patient reports at week 52 from treatment initiation.

### Demographics and Clinical Characteristics

We retrieved data on disease activity assessed with the Systemic Lupus Erythematosus Disease Activity Index 2000 (SLEDAI-2K) (19), and data on organ damage assessed with the Systemic Lupus International Collaborating Clinics (SLICC)/ACR Damage Index (SDI) (20).

Patients were stratified into four ancestry groups based on self-reports, i.e., Asian, Black/African American, Indigenous American and White/Caucasian. Additionally, they were stratified into Hispanics and non-Hispanics, as well as subgroups based on their country of residence, as detailed in Supplementary Table 1.

### Evaluation of HRQoL

HRQoL was assessed utilising the generic instruments Medical Outcomes Study Short Form 36 (SF-36) health survey (21) and Functional Assessment of Chronic Illness Therapy (FACIT) Fatigue (FACIT-F) scale (22). Importantly, the psychometric properties of these two instruments have been reviewed in compliance with the US Food and Drug Administration (FDA) (23) under the auspices of the Outcome Measures in Rheumatology (OMERACT) SLE working group, and are suggested as secondary endpoints in clinical trials (24).

The SF-36 is a questionnaire used for assessment of HRQoL over the preceding 4 weeks. Computation of patients' responses to 36 questions results in eight subscales, each representing a distinct HRQoL aspect, i.e., physical functioning (PF), role physical (RP), bodily pain (BP), general health (GH), social functioning (SF), vitality (VT), role emotional (RE) and mental health (MH). SF-36 subscale scores were calculated according to the SF-36v2 manual (25), and transformed to generate subscale scores ranging from 0 to 100. Subsequently, the SF-36 subscale scores are weighted into two summary scores, i.e., the physical component summary (PCS) and mental component summary (MCS). The component summary scores are norm-based, with a mean of 50 and a standard deviation of 10. All subscales contribute to the derivation of PCS and MCS, albeit differently weighted in each one of them. PF, RP, BP, and GH are referred to as the physical aspects, and SF, VT, RE, and MH are referred to as the mental aspects of SF-36. Higher scores in SF-36 items are interpreted as better HRQoL perceptions. As suggested in literature, we determined the minimal clinically important difference (MCID) for PCS and MCS as scores ≥2.5 and for SF-36 subscales as scores ≥5.0 (26). After management of missing values, all SRI-4 responders had available SF-36 registrations at week 52 (*N* = 760).

The FACIT-F is a survey that evaluates the level of fatigue over the preceding seven days (22). Patient responses to the 13 items of FACIT-F are transformed into a score ranging from 0 (maximal fatigue) to 52 (minimal fatigue). After management of missing values, the number of SRI-4 responders with available FACIT-F registrations at week 52 was 745.

### Definition of Adverse HRQoL

To our knowledge, no established definitions of adverse HRQoL outcomes based on specific cut-offs in SF-36 subscale and component summary scores exist for SLE. We created a US population-based reference group, pairwise matched for age and sex with the BLISS study participants, using normative data from the SF-36 health survey user manual (27, 28).

First, we compared the mean SF-36 subscale and component summary scores of SRI-4 responders with the corresponding scores as derived from the age- and sex-matched norms. Next, we determined adverse HRQoL. In a cohort of patients with RA, Druce et al. defined severe fatigue as SF-36 VT scores corresponding to the 5th percentile of VT scores derived from a Scottish general population-based reference group that was matched for age and sex with the patients (29). Following a similar approach, we defined adverse HRQoL outcomes in SF-36 as subscale or component summary scores equal to or less than the normative 5th percentile (NP5), as derived from the reference group described above. Following this process, the NP5 for each SF-36 scale yielded the following values, as previously reported by our group (30): PF ≤ 52.5; RP ≤ 29.8; BP ≤ 38.6; GH ≤ 41.0; VT ≤ 25.4; SF ≤ 46.2; RE ≤ 26.6; MH ≤ 43.4; PCS ≤ 36.0; MCS ≤ 34.5.

FACIT-F values <30 represent severe fatigue (22), and herein designated adverse FACIT-F outcome.

### Statistics

Data are presented as number (percentage) or mean ± standard deviation (SD) and, in case of non-normal distributions, the median and interquartile range (IQR) are indicated. Pearson's chi-square or Fisher's exact tests were used to investigate associations between dichotomous variables. Comparisons of continuous data between SLE patients and age- and sex-matched norms, as well as comparisons between baseline and week 52, were performed using the Wilcoxon signed-rank test. Comparisons of continuous data between unrelated groups were conducted using the Mann-Whitney *U* test, and across more than two groups using the Kruskal-Wallis test. Multivariable logistic regression models were created in order to assess independence and priority of potential factors associated with adverse HRQoL outcomes.

*P*-values < 0.05 were considered statistically significant. Missing data were imputed using the last observation carried forward (LOCF) or next observation carried backward (NOCB) methods as appropriate. The IBM SPSS version 25 software (IBM Corp., NY, USA) was used for statistical analysis.

### Ethics

Data from the BLISS trials were made available by GlaxoSmithKline (Uxbridge, UK) through the Clinical Study Data Request consortium. The BLISS-52 and BLISS-76 study protocols were approved by regional ethics review boards for all participating centres, and the protocol of the present study was approved by the Swedish Ethical Review Authority (2019-05498).

## Results

### Demographics and Clinical Characteristics

Demographics and clinical characteristics of the SRI-4 responders (*N* = 760) are presented in Table 1. They were mainly women (94.3%) of middle-age (37.3 ± 11.4 years) with a median disease duration of 3.9 (IQR: 1.2–8.5) years. The most represented ancestries were White/Caucasian (46.2%) and Indigenous American (27.6%), whereas 42.0% of the patients reported Hispanic ethnicity. The mean SLEDAI-2K score at week 52 was 3.8 (±2.9), and 453 patients (59.6%) had zero SDI scores (IQR: 0–1).

**Table 1:** Demographics and clinical characteristics of SRI-4 responders in the pooled BLISS study population.

	**SRI-4 responders *N* = 760**	**Hispanics *N* = 319**	**Non-Hispanics *N* = 441**	***P*-value**
**Patient characteristics**			
Age at baseline (years)	37.3 ± 11.4	36.5 ± 10.6	37.9 ± 11.9	0.170
Female sex	717 (94.3%)	302 (94.7%)	415 (94.1%)	0.739
Ancestries
Asian	144 (18.9%)	0	144 (32.7%)	** <0.001**
Black/African American	55 (7.2%)	99 (31.0%)	252 (57.1%)	**<0.001**
Indigenous American^*^	210 (27.6%)	15 (4.7%)	40 (9.1%)	**0.022**
White/Caucasian	351 (46.2%)	205 (64.3%)	5 (1.1%)	**<0.001**
**Clinical data**			
SLE duration at baseline (years)	5.8 (1.2–8.5)	5.1 (1.0–7.8)	6.3 (1.4–9.0)	**0.017**
SLEDAI-2K score
Baseline	10.7 ± 3.6	10.6 ± 3.5	10.7 ± 3.6	0.888
Week 52	3.8 ± 2.9	3.6 ± 2.7	3.9 ± 3.0	0.332
SDI score
Baseline	0.7 ± 1.1 0.0 (0.0–1.0)	0.5 ± 1.0 0.0 (0.0–1.0)	0.8 ± 1.2 0.0 (0.0–1.0)	**<0.001**
Week 52	0.7 ± 1.2 0.0 (0.0–1.0)	0.5 ± 1.0 0.0 (0.0–1.0)	0.9 ± 1.3 0.0 (0.0–1.0)	**<0.001**
SDI score > 0
Baseline	293 (38.6%)	90 (28.2%)	203 (46.0%)	**<0.001**
Week 52	307 (40.4%)	96 (30.1%)	211 (47.8%)	**<0.001**
Serological profile at baseline
Anti-dsDNA (+)	517 (68.0%)	216 (67.7%)	301 (68.3%)	0.874
Anti-Sm (+)	224 (29.6%); N = 758	109 (34.2%)	115 (26.2%); N = 439	**0.018**
Low C3	311 (40.9%)	122 (38.2%)	189 (42.9%)	0.202
Low C4	395 (52.0%)	165 (51.7%)	230 (52.2%)	0.907
Prednisone eq. dose (mg/day)
Baseline	11.7 ± 9.0	12.5 ± 9.1	11.1 ± 9.0	**0.028**
Week 52	8.7 ± 6.8; *N* = 754	9.4 ± 7.2; *N* = 318	8.2 ± 6.6; *N* = 436	**0.022**
Antimalarial agents at week 52^†^	478 (62.9%)	223 (69.9%)	255 (57.8%)	**0.001**
Immunosuppressants at week 52
Azathioprine	149 (19.6%)	76 (23.8%)	73 (16.6%)	**0.013**
Methotrexate	78 (10.3%)	36 (11.3%)	42 (9.5%)	0.430
Mycophenolic acid	72 (9.5%)	25 (7.8%)	47 (10.7%)	0.190
Other immunosuppressants^‡^	15 (2.0%)	6 (1.9%)	9 (2.0%)	0.876
Trial intervention
Placebo	217 (28.6%)	95 (29.8%)	122 (27.7%)	0.524
Belimumab 1 mg/kg	258 (33.9%)	113 (35.4%)	145 (32.9%)	0.465
Belimumab 10 mg/kg	285 (37.5%)	111 (34.8%)	174 (39.5%)	0.190

*Data are presented as numbers (percentage) or means ± standard deviation. In case of non-normal distributions, medians (interquartile range) are indicated. In case of missing values, the total number of patients with available data is indicated. Statistically significant P-values are in bold*.

* *Alaska Native or American Indian from North, South or Central America*.

† *Hydroxychloroquine, chloroquine, mepacrine, mepacrine hydrochloride or quinine sulphate*.

‡ *Cyclosporine, oral cyclophosphamide, leflunomide, mizoribine or thalidomide*.

*C3, complement component protein 3; C4, complement component protein 4; dsDNA, double stranded DNA; SDI, Systemic Lupus International Collaborating Clinics (SLICC)/American College of Rheumatology (ACR) Damage Index; SLE, systemic lupus erythematosus; SLEDAI-2K, SLE Disease Activity Index 2000; Sm, Smith; SRI-4, SLE Responder Index 4*.

### HRQoL Outcome at Week 52 of Treatment

**Figure 1:**
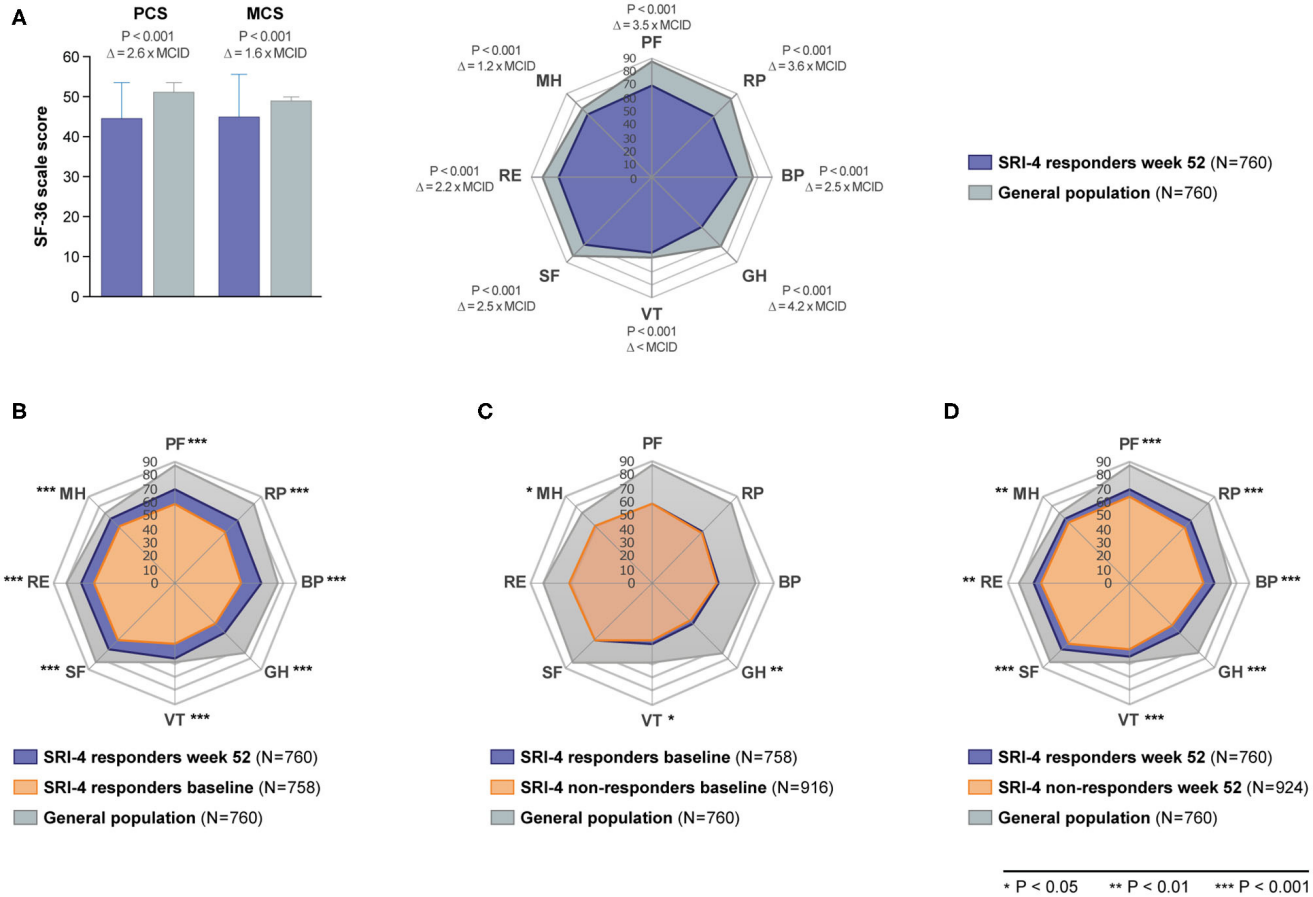
SF-36 scores among SRI-4 responders and non-responders before and after trial intervention. The bar chart **(A)** illustrates comparisons of SF-36 component summary scores between SRI-4 responders at week 52 and age- and sex-matched US population-based norms. The height of the bars represents mean scores, while the height of the whiskers above the bars represents standard deviation. The radial charts illustrate comparisons of mean SF-36 subscale scores between **(A)** SRI-4 responders at week 52 and age- and sex-matched US population-based norms, **(B)** baseline and week 52 in SRI-4 responders, **(C)** SRI-4 responders and non-responders at baseline, and **(D)** SRI-4 responders and non-responders at week 52. Asterisks indicate statistically significant associations. BP, bodily pain; GH, general health; MCID, minimal clinically important difference; MCS, mental component summary; MH, mental health; NP5, normative 5th percentile; PCS, physical component summary; PF, physical functioning; RE, role emotional; RP, role physical; SF, social functioning; SF-36, short form 36 health survey; SLE, systemic lupus erythematosus; VT, vitality; Δ, delta (difference).

**Figure 2:**
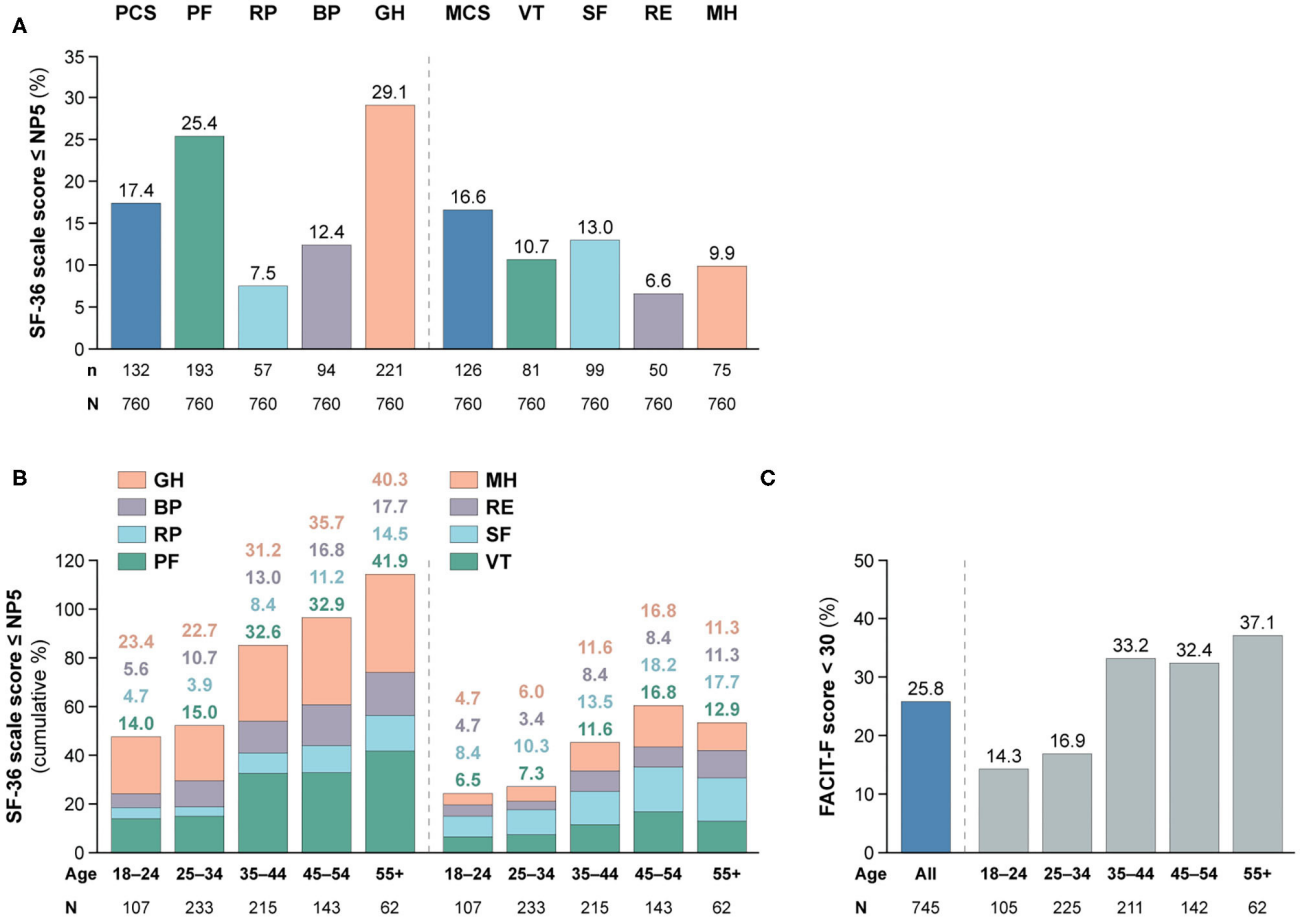
Adverse HRQoL outcome despite clinical improvement. This figure illustrates the prevalence of adverse HRQoL outcomes. Panel **(A)** shows frequencies of adverse HRQoL outcome defined as SF-36 physical and mental scale scores ≤NP5, and panel **(B)** shows their cumulative frequencies within different age categories. Panel **(C)** shows frequencies of FACIT-F scores <30, including frequencies within different age categories. BP, bodily pain; FACIT-F, Functional Assessment of Chronic Illness Therapy–Fatigue; GH, general health; MCS, mental component summary; MH, mental health; n, number of patients reporting adverse HRQoL outcomes; N, total number of patients with available data; NP5, normative 5th percentile; PCS, physical component summary; PF, physical functioning; RE, role emotional; RP, role physical; SF, social functioning; SF-36, short form 36 health survey; SLE, systemic lupus erythematosus; VT, vitality.

As illustrated in Figure 1, patients with SLE who achieved SRI-4 response reported worse HRQoL at week 52 from treatment initiation than US population-based norms individually matched for age and sex (*P* < 0.001 for all SF-36 scales), which exceeded the MCID in all SF-36 items but VT (Figures 1A,B; Supplementary Table 2). The differences were most prominent for GH, RP and PF, yielding 4.2, 3.6, and 3.5 times the MCID lower mean scores than the matched norms, respectively. Figure 2 delineates proportions of SRI-4 responders who reported adverse HRQoL outcomes at week 52 from treatment initiation. Overall, proportions of patients experiencing adverse HRQoL outcomes were higher within physical vs. mental aspects, with GH (29.1%) and PF (25.4%) being the SF-36 domains yielding the highest frequencies (Figure 2A). SF-36 VT scores ≤ NP5 were reported by 10.7% of SRI-4 responders, whereas 25.8% reported FACIT-F scores <30. As expected, frequencies of adverse HRQoL outcome at week 52 were lower in SRI-4 responders compared with non-responders with regard to all SF-36 items and FACIT-F score <30 (*P* < 0.001 for all; Supplementary Table 3), with the greatest absolute difference observed for SF-36 GH ≤ NP5 (29.1% vs. 47.2%). Moreover, SRI-4 responders displayed improvements in all SF-36 subscale scores from baseline through week 52 (Figure 1B); while they generally reported similar scores to non-responders at baseline (Figure 1C), they scored higher in all subscales at week 52 (Figure 1D). Consequently, SRI-4 responders reported lower proportions of adverse HRQoL at week 52 compared with baseline (*P* < 0.001 for all SF-36 items and FACIT-F; Supplementary Table 4).

### Adverse HRQoL Outcomes Across Age Categories

We observed gradually higher frequencies of adverse HRQoL outcomes within SF-36 physical subscales with increasing age category (Figure 2B); of 62 patients aged 55+ years, 26 (41.9%) reported adverse PF and 25 (40.3%) reported adverse GH. The frequency of adverse HRQoL outcomes within SF-36 mental subscales also increased with age, but peaked in the category of 45–54 years. We found greater proportions of patients reporting FACIT-F scores <30 within higher age categories, which however plateaued from 35 years of age (Figures 2B,C).

### Comparisons Across Ancestries

**Figure 3:**
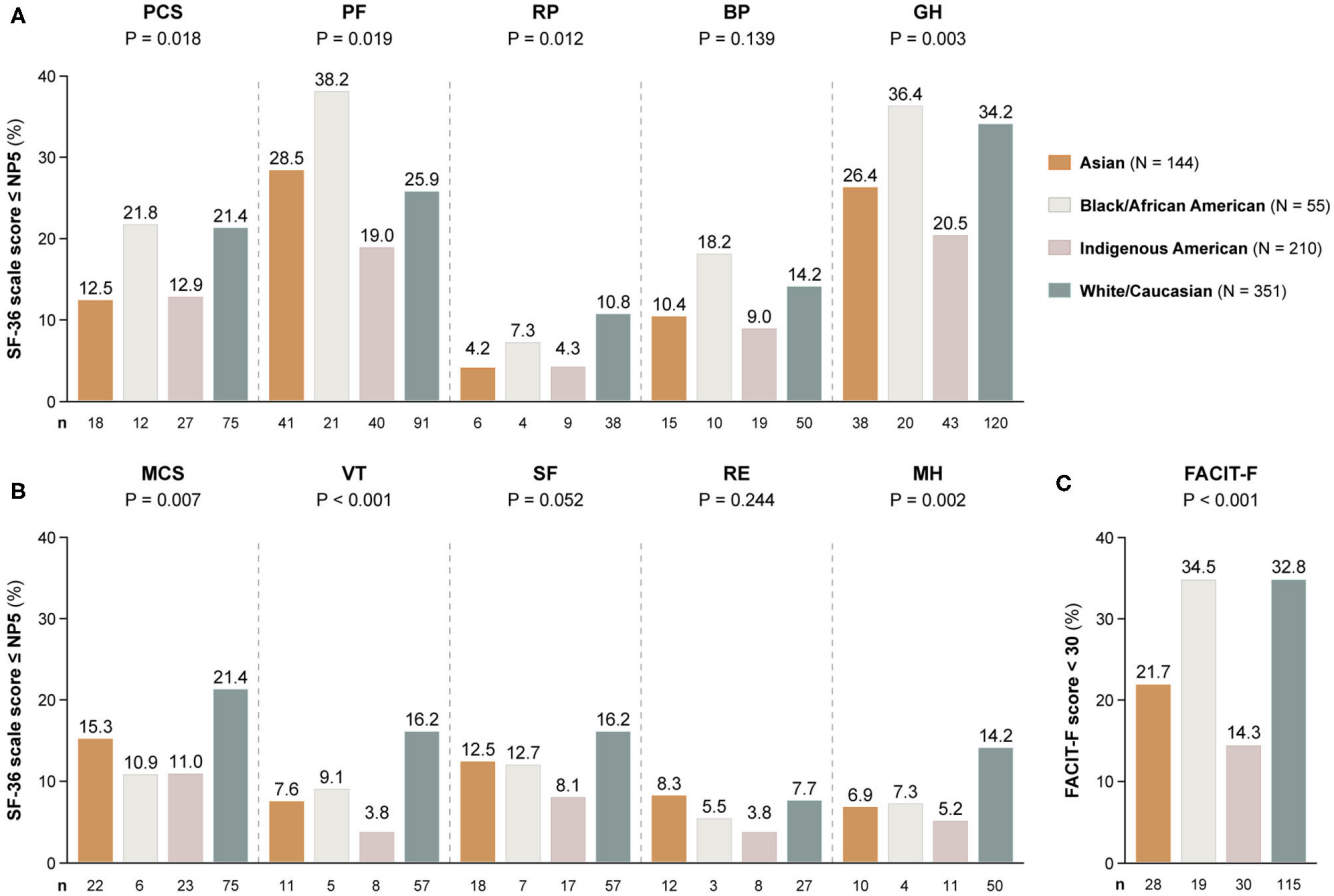
Adverse HRQoL outcome despite clinical improvement across different ancestries. This figure illustrates comparisons of the prevalence of adverse HRQoL outcomes at week 52 from treatment initiation across patients with SLE of Asian, Black/African American, Indigenous American, and White/Caucasian ancestries. **(A,B)** Delineate proportions of patients reporting SF-36 scores below or equal to the 5th percentile of the corresponding scores as derived from a general population of the same age and sex, within physical and mental aspects, respectively. **(C)** Delineates proportions of patients reporting FACIT-F scores <30, signifying severe fatigue. The number of patients reporting adverse HRQoL outcomes (*n*) is indicated below the respective bar. *P*-values derived from chi-square tests. BP, bodily pain; FACIT-F, Functional Assessment of Chronic Illness Therapy-Fatigue; GH, general health; MCS, mental component summary; MH, mental health; NP5, normative 5th percentile; PCS, physical component summary; PF, physical functioning; RE, role emotional; RP, role physical; SF, social functioning; SF-36, short form 36 health survey; VT, vitality.

Table 2 shows demographics and clinical characteristics of the study participants stratified by their ancestry. As delineated in Figure 3, proportions of patients with adverse SF-36 PCS differed across ancestries (*P* = 0.018); Black/African Americans showed the highest frequency (21.8%), followed by White/Caucasians (21.4%), while Asian and Indigenous American patients reported the lowest frequencies (12.5 and 12.9%, respectively). Similar patterns were observed for adverse PF (*P* = 0.019) and GH (*P* = 0.003), within which Black/African Americans reported the highest (38.2 and 36.4%, respectively) and Indigenous Americans the lowest (19.0 and 20.5%, respectively) frequencies.

**Table 2:** Demographics and clinical characteristics of patients across different ancestries.

	**Asian *N* = 144**	**Black/ African American *N* = 55**	**Indigenous American *N* = 210**	**White/ Caucasian *N* = 351**	***P*-value**
**Patient characteristics**					
Age at baseline (years)	32.7 ± 9.4	38.5 ± 11.9	36.7 ± 10.4	39.4 ± 12.0	**<0.001**
Female sex	136 (94.4%)	53 (96.4%)	201 (95.7%)	327 (93.2%)	0.556
**Clinical data**					
SLE duration at baseline (years)	5.0 (0.7–7.5)	6.5 (1.2–9.0)	4.7 (0.9–6.8)	6.7 (1.6–10.4)	**0.002**
SLEDAI-2K score
Baseline	11.3 ± 3.6	10.3 ± 3.3	10.6 ± 3.5	10.5 ± 3.6	0.060
Week 52	4.5 ± 3.0	3.6 ± 2.6	3.6 ± 2.8	3.7 ± 2.9	0.260
SDI score
Baseline	0.5 ± 1.0 0.0 (0.0–1.0)	0.9 ± 1.3 0.0 (1.0–1.0)	0.4 ± 0.9 0.0 (0.0–1.0)	0.8 ± 1.3 0.0 (0.0–1.0)	**<0.001**
Week 52	0.6 ± 1.0 0.0 (0.0–1.0)	1.0 ± 1.5 0.0 (1.0–1.0)	0.4 ± 0.9 0.0 (0.0–1.0)	0.9 ± 1.3 0.0 (0.0–1.0)	**<0.001**
SDI score > 0
Baseline	48 (33.3%)	28 (50.9%)	54 (25.7%)	163 (46.4%)	**<0.001**
Week 52	50 (34.7%)	29 (52.7%)	57 (27.1%)	171 (48.7%)	**<0.001**
Serological profile at baseline
Anti-dsDNA (+)	122 (84.7%)	37 (67.3%)	141 (67.1%)	217 (61.8%)	**<0.001**
Anti-Sm (+)	55 (38.2%)	22 (40.0%)	74 (35.2%)	73 (20.9%)	**<0.001**
Low C3	88 (61.1%)	18 (32.7%)	86 (41.0%)	119 (33.9%)	**<0.001**
Low C4	85 (59.0%)	17 (30.9%)	113 (53.8%)	180 (51.3%)	**0.005**
Prednisone eq. dose (mg/day)
Baseline	13.3 ± 9.3	12.6 ± 10.0	12.0 ± 8.2	10.7 ± 9.2	0.236
Week 52	8.4 ± 5.1; N = 143	9.9 ± 8.7; N = 54	9.3 ± 6.7; N = 209	8.3 ± 7.2; N = 348	**0.010**
Antimalarial agents at week 52^†^	83 (57.6%)	35 (63.6%)	147 (70.0%)	213 (60.7%)	0.072
Immunosuppressants at week 52
Azathioprine	23 (16.0%)	11 (20.0%)	54 (25.7%)	61 (17.4%)	0.063
Methotrexate	4 (2.8%)	7 (12.7%)	30 (14.3%)	37 (10.5%)	**0.005**
Mycophenolic acid	11 (7.6%)	9 (16.4%)	14 (6.7%)	38 (10.8%)	0.098
Other immunosuppressants^‡^	2 (1.4%)	2 (3.6%)	3 (1.4%)	8 (2.3%)	0.675
Trial intervention
Placebo	41 (28.5%)	22 (40.0%)	61 (29.0%)	93 (26.5%)	0.232
Belimumab 1 mg/kg	44 (30.6%)	15 (27.3%)	74 (35.2%)	125 (35.6%)	0.490
Belimumab 10 mg/kg	59 (41.0%)	18 (32.7%)	75 (35.7%)	133 (37.9%)	0.663

*Data are presented as numbers (percentage) or means ± standard deviation. In case of non-normal distributions, medians (interquartile range) are indicated. In case of missing values, the total number of patients with available data is indicated. Statistically significant P-values are in bold*.

* *Alaska Native or American Indian from North, South or Central America*.

† *Hydroxychloroquine, chloroquine, mepacrine, mepacrine hydrochloride or quinine sulphate*.

‡ *Cyclosporine, oral cyclophosphamide, leflunomide, mizoribine or thalidomide*.

*C3, complement component protein 3; C4, complement component protein 4; dsDNA, double stranded DNA; SDI, Systemic Lupus International Collaborating Clinics (SLICC)/American College of Rheumatology (ACR) Damage Index; SLE, systemic lupus erythematosus; SLEDAI-2K, SLE Disease Activity Index 2000; Sm, Smith*.

Within the SF-36 mental scales, proportions of patients reporting adverse HRQoL outcomes differed regarding MCS (*P* = 0.007), VT (*P* < 0.001) and MH (*P* = 0.002). With regard to these three SF-36 scales, White/Caucasians showed the highest frequencies (21.4, 16.2, and 14.2%, respectively), whereas Indigenous Americans reported the lowest frequencies in all SF-36 mental subscales, i.e., 8.1% within SF, 5.2% within MH and 3.8% within VT and RE (Figure 3).

### Comparisons Across Country Groups

**Figure 4:**
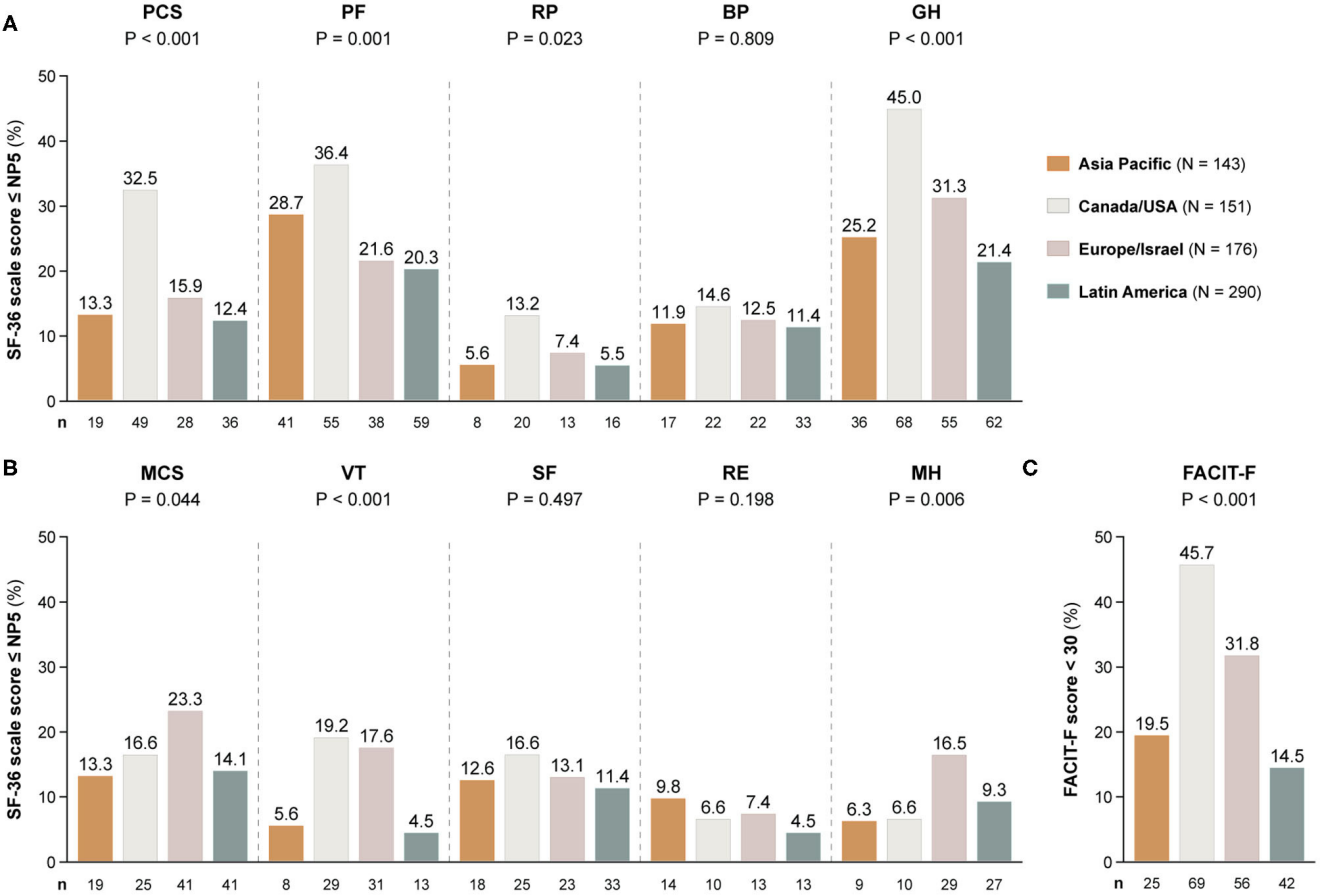
Adverse HRQoL outcome despite clinical improvement across different country groups. This figure illustrates comparisons of the prevalence of adverse HRQoL outcomes at week 52 from treatment initiation across patients with SLE from Asia Pacific, Canada/USA, Europe, and Latin America. **(A,B)** Delineate proportions of patients reporting SF-36 scores below or equal to the 5th percentile of the corresponding scores as derived from a general population of the same age and sex, within physical and mental aspects, respectively. **(C)** Delineates proportions of patients reporting FACIT-F scores <30, signifying severe fatigue. The number of patients reporting adverse HRQoL outcomes (*n*) is indicated below the respective bar. *P*-values derived from chi-square tests. BP, bodily pain; FACIT-F, Functional Assessment of Chronic Illness Therapy-Fatigue; GH, general health; MCS, mental component summary; MH, mental health, NP5, normative 5th percentile; PCS, physical component summary; PF, physical functioning; RE, role emotional; RP, role physical; SF, social functioning; SF-36, short form 36 health survey; VT, vitality.

A similar analysis, albeit stratifying the patients by country groups, is presented in Figure 4. The demographics and clinical characteristics of these groups are shown in Table 3.

**Table 3:** Demographics and clinical characteristics of patients across different country groups.

	**Asia Pacific *N* = 143**	**Canada/USA *N* = 151**	**Europe^#^*N* = 176**	**Latin America *N* = 290**	***P*-value**
**Patient characteristics**					
Age at baseline (years)	32.9 ± 9.9	41.7 ± 11.6	38.1 ± 12.0	36.7 ± 10.7	**<0.001**
Female sex	135 (94.4%)	139 (92.1%)	167 (94.9%)	276 (95.2%)	0.582
Ancestries
Asian	138 (96.5%)	5 (3.3%)	1 (0.6%)	0	**<0.001**
Black/African American	0	39 (25.8%)	1 (0.6%)	15 (5.2%)	**<0.001**
Indigenous American^*^	1 (0.7%)	8 (5.3%)	1 (0.6%)	200 (69.0%)	**<0.001**
White/Caucasian	4 (2.8%)	99 (65.6%)	173 (98.3%)	75 (25.9%)	**<0.001**
Hispanic ethnicity	0	34 (22.5%)	0	285 (98.3%)	**<0.001**
**Clinical data**					
SLE duration at baseline (years)	3.6 (0.8–7.4)	4.9 (1.4–9.6)	5.0 (1.7–10.3)	3.2 (1.0–7.3)	**0.004**
SLEDAI-2K score
Baseline	11.4 ± 3.6	10.3 ± 3.4	10.5 ± 3.8	10.6 ± 3.5	**0.016**
Week 52	3.8 ± 2.3	3.5 ± 2.5	3.8 ± 3.2	3.3 ± 2.4	0.074
SDI score					
Baseline	0.5 ± 0.9 0.0 (0.0–1.0)	1.1 ± 1.5 0.0 (1.0–1.0)	0.7 ± 1.1 0.0 (0.0–1.0)	0.5 ± 1.0 0.0 (0.0–1.0)	**<0.001**
Week 52	0.6 ± 1.0 0.0 (0.0–1.0)	1.2 ± 1.6 0.0 (0.0–2.0)	0.8 ± 1.1 0.0 (0.0–1.0)	0.5 ± 1.0 0.0 (0.0–1.0)	**<0.001**
SDI score > 0
Baseline	47 (32.9%)	87 (57.6%)	75 (42.6%)	84 (29.0%)	**<0.001**
Week 52	49 (34.3%)	89 (58.9%)	79 (44.9%)	90 (31.0%)	**<0.001**
Serological profile at baseline
Anti-dsDNA (+)	119 (83.2%)	80 (53.0%)	122 (69.3%)	196 (67.6%)	**<0.001**
Anti-Sm (+)	53 (37.1%)	36 (23.8%)	36 (20.7%); N=174	99 (34.1%)	**0.001**
Low C3	86 (60.1%)	45 (29.8%)	71 (40.3%)	109 (37.6%)	**<0.001**
Low C4	85 (59.4%)	55 (36.4%)	104 (59.1%)	151 (52.1%)	**<0.001**
Prednisone eq. dose (mg/day)
Baseline	13.1 ± 9.2	7.1 ± 8.4	12.1 ± 8.0	13.1 ± 9.1	**<0.001**
Week 52	8.4 ± 5.2; N=142	5.3 ± 6.2; N=149	10.0 ± 7.0; N=174	9.9 ± 7.2; N=289	**<0.001**
Antimalarial agents at week 52^†^	85 (59.4%)	107 (70.9%)	84 (47.7%)	202 (69.7%)	**<0.001**
Immunosuppressants at week 52
Azathioprine	22 (15.4%)	22 (14.6%)	33 (18.8%)	72 (24.8%)	**0.027**
Methotrexate	6 (4.2%)	24 (15.9%)	13 (7.4%)	35 (12.1%)	**0.004**
Mycophenolic acid	8 (5.6%)	23 (15.2%)	24 (13.6%)	17 (5.9%)	**0.001**
Other immunosuppressants^‡^	3 (2.1%)	3 (2.0%)	3 (1.7%)	6 (2.1%)	0.993
Trial intervention
Placebo	42 (29.4%)	45 (29.8%)	42 (23.9%)	88 (30.3%)	0.472
Belimumab 1 mg/kg	42 (29.4%)	59 (39.1%)	57 (32.4%)	100 (34.5%)	0.343
Belimumab 10 mg/kg	59 (41.3%)	47 (31.1%)	77 (43.8%)	102 (35.2%)	0.069

*Data are presented as numbers (percentage) or means ± standard deviation. In case of non-normal distributions, medians (interquartile range) are indicated. In case of missing values, the total number of patients with available data is indicated. Statistically significant P-values are in bold*.

* *Alaska Native or American Indian from North, South or Central America*.

† *Hydroxychloroquine, chloroquine, mepacrine, mepacrine hydrochloride or quinine sulphate*.

‡ *Cyclosporine, oral cyclophosphamide, leflunomide, mizoribine or thalidomide*.

# *Including Israel*.

*C3, complement component protein 3; C4, complement component protein 4; dsDNA, double stranded DNA; SDI, Systemic Lupus International Collaborating Clinics (SLICC)/American College of Rheumatology (ACR) Damage Index; SLE, systemic lupus erythematosus; SLEDAI-2K, SLE Disease Activity Index 2000; Sm, Smith*.

Patients residing in Canada/USA most frequently reported adverse outcome in all physical SF-36 items (13.2–45.0%), whereas patients from Latin America showed the lowest frequencies (5.5–21.4%). Within mental HRQoL aspects, patients residing in Canada/USA and Europe/Israel most frequently reported adverse VT (19.2 and 17.6%, respectively) and FACIT-F (45.7 and 31.8%, respectively), and the highest frequencies of adverse MH were seen among patients from Europe/Israel (15.5%).

### Comparisons Between Hispanics and Non-hispanics

Patients of Hispanic/Latin American ethnicity had shorter disease duration than non-Hispanics (median; IQR: 5.1; 1.0–7.8 vs. 6.3; 1.4–9.0 years; *P* = 0.017), and fewer patients among Hispanics had SDI scores >0 at baseline (28.2 vs. 46.0%; *P* < 0.001) and week 52 (30.1 vs. 47.8%; *P* < 0.001). At week 52, Hispanics were on slightly higher mean prednisone or prednisone equivalent doses (9.4 ± 7.2 vs. 8.2 ± 6.6 mg/day; *P* = 0.022), and a higher percentage among them were on antimalarial agents (69.9 vs. 57.8%; *P* = 0.001; Table 1).

**Figure 5:**
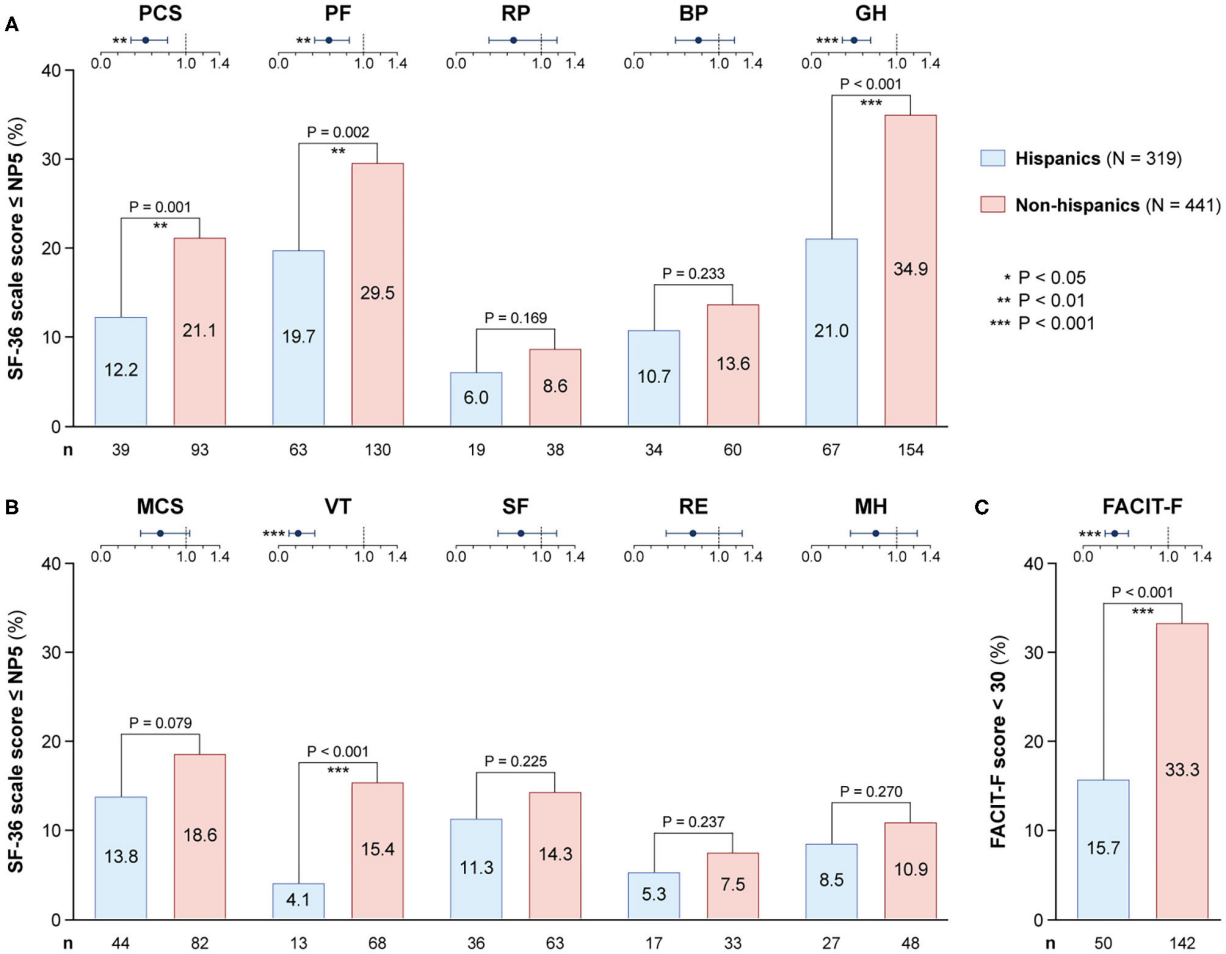
Adverse HRQoL outcome despite clinical improvement in Hispanics versus non-Hispanics. This figure illustrates comparisons of the prevalence of adverse HRQoL outcomes at week 52 from treatment initiation between patients with SLE of Hispanic/Latin American and non-Hispanic ethnicity. **(A,B)** Delineate proportions of patients reporting SF-36 scores below or equal to the 5th percentile of the corresponding scores as derived from a general population of the same age and sex, within physical and mental aspects, respectively. **(C)** Delineates proportions of patients reporting FACIT-F scores <30, signifying severe fatigue. In the forest plots, circles designate the corresponding unadjusted odds ratios and whiskers designate 95% confidence intervals. The number of patients reporting adverse HRQoL outcomes (*n*) is indicated below the respective bar. *P*-values derived from chi-square tests. Significant *P*-values are indicated with asterisks. BP, bodily pain; FACIT-F, Functional Assessment of Chronic Illness Therapy-Fatigue; GH, general health; MCS, mental component summary; MH, mental health; NP5, normative 5th percentile; PCS, physical component summary; PF, physical functioning; RE, role emotional; RP, role physical; SF, social functioning; SF-36, short form 36 health survey; VT, vitality.

As seen in Figure 5, frequencies of patients reporting adverse HRQoL outcomes in the physical domains of SF-36 were lower in Hispanics vs. non-Hispanics regarding PCS (12.2 vs. 21.1%; odds ratio, OR: 0.52; 95% confidence interval, CI: 0.35–0.58; *P* = 0.001), PF (19.7 vs. 29.5%; OR: 0.59; 95% CI: 0.42–0.83; *P* = 0.002) and GH (21.0 vs. 34.9%; OR: 0.50; 95% CI: 0.36–0.69; *P* < 0.001). With regard to the mental compartment of SF-36, a lower proportion of Hispanic patients reported adverse VT (4.1%) compared with non-Hispanics (15.4%; OR: 0.23; 95% CI: 0.13–0.43; *P* < 0.001). Likewise, the proportion of patients with FACIT-F scores <30 was lower among Hispanics (15.7 vs. 33.3%; OR: 0.37; 95% CI: 0.26–0.53; *P* < 0.001).

### Factors Associated With Adverse HRQoL

First, we compared demographic and clinical characteristics of patients with adverse vs. non-adverse PCS, MCS and FACIT-F at week 52. Compared with those reporting adverse PCS, individuals with non-adverse PCS were younger and had lower SDI scores (Supplementary Table 5). Higher proportions of anti-dsDNA positive (70.7 vs. 55.3%; *P* = 0.001) patients as well as patients with low C3 (43.6 vs. 28.0%; *P* = 0.001) and low C4 (54.8 vs. 38.6%; *P* = 0.001) levels at baseline were seen among patients who reported non-adverse PCS at week 52. Notably, a higher proportion of anti-Sm positive patients was seen within patients who reported non-adverse MCS (31.5 vs. 19.8%; *P* = 0.009; Supplementary Table 6). Patients with FACIT-F scores ≥30 at week 52 were younger and had lower SDI scores compared with patients with severe fatigue (Supplementary Table 7). Moreover, more patients within the non-severe fatigue group were anti-dsDNA (71.4 vs. 57.8%; *P* < 0.001) and anti-Sm (32.7 vs. 20.8%; *P* = 0.002) positive at baseline, and more had been treated with belimumab 10 mg/kg (39.8 vs. 29.2%; *P* = 0.009).

Subsequently, we created multivariable logistic regression models to assess independence and account for confounding potentiality. Covariates in the models included age, sex, ancestry, Hispanic ethnicity, SLEDAI-2K and SDI scores at week 52, and the trial intervention, i.e., belimumab 10 mg/kg or 1 mg/kg with placebo as the reference comparator.

**Figure 6:**
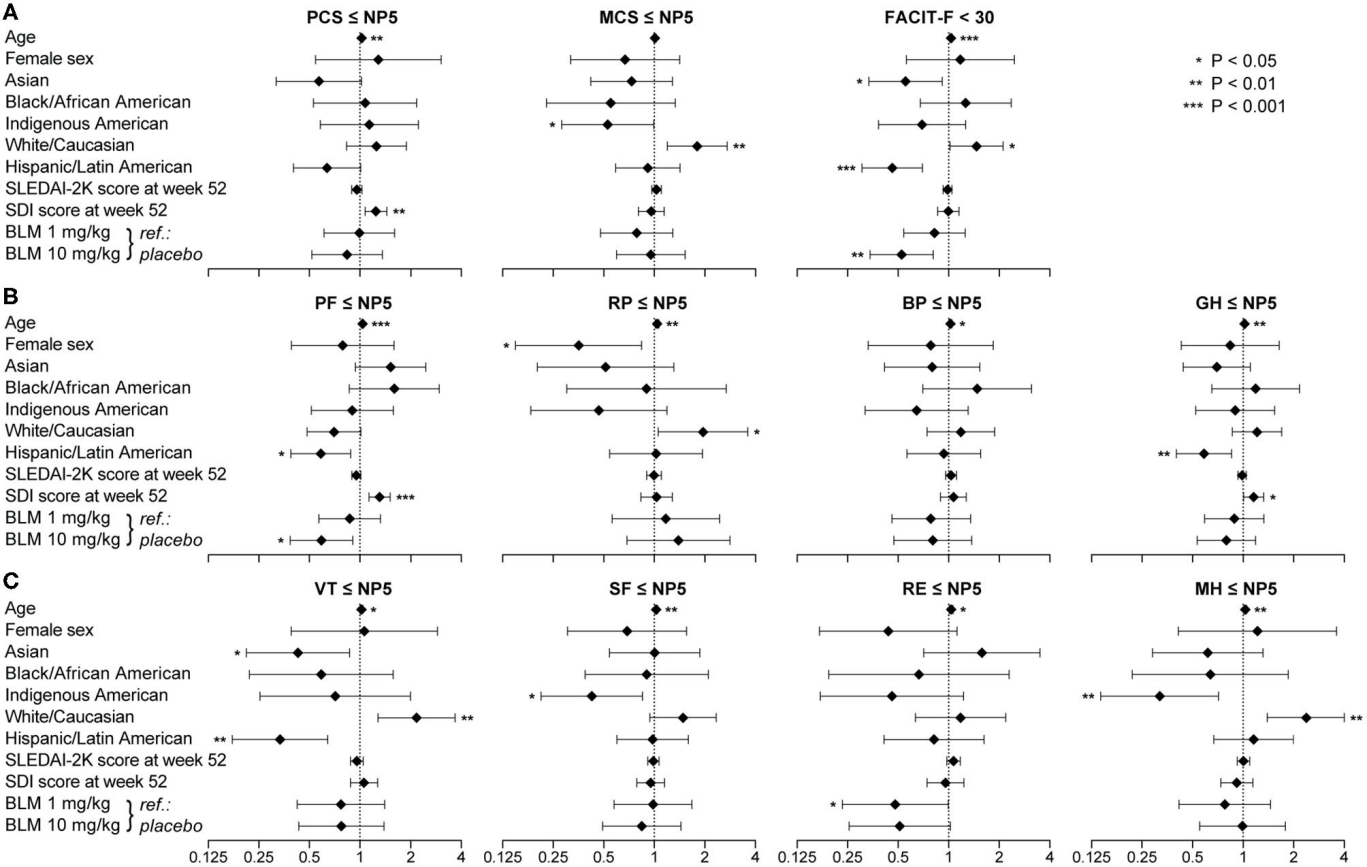
Factors associated with adverse HRQoL outcomes. The forest plots illustrate results from multivariable logistic regression models, with the SF-36 component summary scores ≤NP5 **(A)**, physical subscale scores ≤NP5 **(B)**, mental subscale scores ≤NP5 **(C)** and FACIT-F scores <30 **(A)** at week 52 from treatment initiation as the dependant variables. Diamonds represent odds ratios and whiskers represent 95% confidence intervals. Asterisks indicate statistically significant associations. BP, bodily pain; CI, confidence interval; FACIT-F, Functional Assessment of Chronic Illness Therapy–Fatigue; GH, general health; MCS, mental component summary; MH, mental health; NP5, normative 5th percentile; OR, odds ratio; PCS, physical component summary; PF, physical functioning; RE, role emotional; RP, role physical; SF, social functioning; SF-36, short form 36 health survey; VT, vitality.

Increasing age was associated with adverse HRQoL outcome in all physical and mental SF-36 scales, except for MCS, and with adverse FACIT-F (Figure 6). White/Caucasian ancestry was associated with adverse RP (OR: 1.95; 95% CI: 1.06–3.59; *P* = 0.033), VT (OR: 2.17; 95% CI: 1.28–3.68; *P* = 0.004) and MH (OR: 2.37; 95% CI: 1.39–4.05; *P* = 0.002), as well as with FACIT-F scores <30 (OR: 1.47; 95% CI: 1.02–2.11; *P* = 0.039). Conversely, Hispanic ethnicity was associated with lower proportions of adverse PF (OR: 0.59; 95% CI: 0.39–0.88; *P* = 0.010), GH (OR: 0.59; 95% CI: 0.40–0.85; *P* = 0.005) and VT (OR: 0.34; 95% CI: 0.18–0.64; *P* = 0.001), as well as with FACIT-F scores <30 (OR: 0.31; 95% CI: 0.46–0.70; *P* < 0.001).

Increasing SDI scores at week 52, representing organ damage accrued from disease onset until the evaluation, were associated with adverse HRQoL outcomes within physical SF-36 scales, including PCS (OR: 1.25; 95% CI: 1.07–1.45; *P* = 0.004), PF (OR: 1.31; 95% CI: 1.13–1.51; *P* < 0.001) and GH (OR: 1.16; 95% CI: 1.01–1.33; *P* = 0.040), but not within mental SF-36 scales or FACIT-F. We found no significant association between SLEDAI-2K scores and adverse HRQoL outcomes (Figure 6). Notably, addition of belimumab 10 mg/kg to ST was associated with lower frequencies of adverse PF (OR: 0.59; 95% CI: 0.39–0.91; *P* = 0.016) and FACIT-F (OR: 0.53; 95% CI: 0.34–0.81; *P* = 0.004). Similar results were seen for age, sex, SLEDAI-2K, SDI and belimumab use when we included country groups instead of ancestries or ethnic origin as covariates in the models (Supplementary Figure 1).

**Figure 7:**
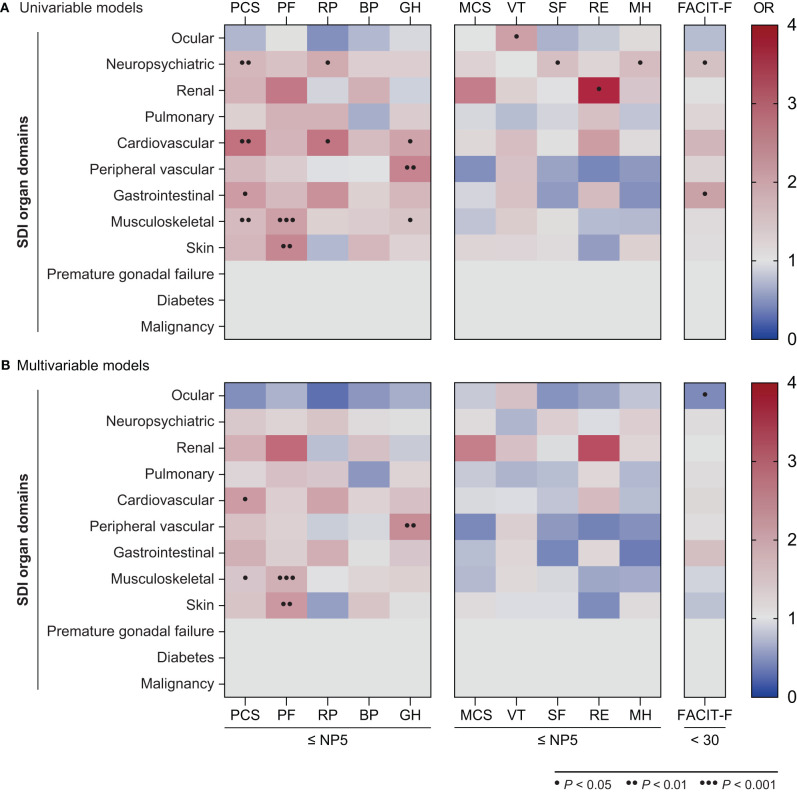
Associations between SDI organ domains and adverse HRQoL outcomes. The heatmaps illustrate ORs deriving from univariable **(A)** and multivariable **(B)** logistic regression models, with the SF-36 scale scores ≤NP5 and FACIT-F scores <30 at week 52 as the dependant variables. Dots indicate statistically significant associations. BP, bodily pain; FACIT-F, Functional Assessment of Chronic Illness Therapy–Fatigue; GH, general health; MCS, mental component summary; MH, mental health; OR, odds ratio; PCS, physical component summary; PF, physical functioning; RE, role emotional; RP, role physical; SF, social functioning; SF-36, short form 36 health survey; VT, vitality.

Next, in order to determine the type of established organ damage accrued from disease onset until the time of evaluation (week 52) that accounted for the observed association between SDI scores and adverse HRQoL outcomes, we created separate univariable and multivariable logistic regression models for each one of the SDI organ domains (Figure 7, Supplementary Tables 8–10). Damage in the neuropsychiatric, cardiovascular, gastrointestinal and musculoskeletal domains was associated with adverse PCS in univariable models; this association remained significant after adjustment for the cardiovascular (OR: 2.12; 95% CI: 1.07–4.21; *P* = 0.032) and musculoskeletal (OR: 1.41; 95% CI: 1.01–1.96; *P* = 0.041) domains. With regard to mental aspects, damage in the neuropsychiatric domain was associated with adverse SF-36 SF (OR: 1.55; 95% CI: 1.01–2.38 *P* = 0.044) and MH (OR: 1.59; 95% CI: 1.00–2.53; *P* = 0.050), whereas renal damage was associated with adverse RE (OR: 3.70; 95% CI: 1.01–13.57; *P* = 0.048) in univariable analyses; however, these associations did not reach statistical significance in the adjusted models. Finally, damage in the neuropsychiatric (OR: 1.56; 95% CI: 1.04–2.16; *P* = 0.031) and gastrointestinal (OR: 2.01; 95% CI: 1.05–3.82; *P* = 0.034) domains was associated with severe fatigue (FACIT-F scores <30) in the univariable but not the multivariable models.

## Discussion

Herein, we investigated frequencies of adverse HRQoL outcome and contributing factors in 760 patients with SLE who showed adequate clinical response to a 52-week long intervention with standard therapy along with belimumab or placebo, using previously reported definitions (30). We observed clinically important diminutions of patient-reported HRQoL in multiple physical, mental and social aspects compared with the general population, and high frequencies of adverse HRQoL outcomes, especially within physical domains of SF-36 and FACIT-F. Overall, higher frequencies of adverse HRQoL outcomes were seen with increasing age. Black/African American and White/Caucasian patients reported higher frequencies of adverse HRQoL outcomes than Asians and Indigenous Americans, while Hispanic/Latin American patients experienced adverse HRQoL less frequently than non-Hispanics. Importantly, addition of the licenced dose of intravenous belimumab to standard therapy was associated with lower frequencies of adverse physical functioning and severe fatigue.

Improvements in multiple HRQoL aspects following treatment with conventional synthetic or biological disease-modifying agents have been documented in patients with SLE, especially along with clinical improvements or attainment of low disease activity or remission (5–9, 31), which we corroborated in the present study, demonstrating that treatment responders improved in all SF-36 items during the study period and reported higher SF-36 scores than non-responders to treatment. However, it is important to emphasise that improvement does not necessarily reflect a satisfactory health state perception. Studies of patients with RA have demonstrated considerable frequencies of persisting pain and severe fatigue among patients who achieved a good clinical response or remission following treatment (12, 13). In light of the above, we studied the prevalence of HRQoL outcome in patients who met the primary endpoint of two phase III clinical trials of belimumab in patients with SLE. First, we observed clinically important diminutions of patient-reported HRQoL in multiple aspects compared with matched US population-based norms. In general health, role physical and physical functioning, these differences yielded 4.2, 3.6 and 3.5 times the MCID lower scores, respectively. Next, despite conservative definitions of adverse HRQoL outcomes, especially for SF-36, we found high frequencies at week 52 among responders. Ranging from 6.6% (for role emotional) to 29.1% (for general health), these frequencies exceeded the expected frequencies as derived from individually matched US population-based norms in all physical and mental aspects.

Frequencies of adverse HRQoL outcome were more prominent within physical compared with mental domains of SF-36, which is consistent with the general trend in SLE patients as derived from real-world SF-36 data (32, 33). Since SLE is a highly heterogeneous disease, the clinical phenotype is expected to impact on HRQoL. For instance, in a cohort of patients with lupus nephritis, those with active disease reported worse HRQoL in mental aspects than patients with inactive disease, but the groups did not differ in physical health or pain (34). Most study participants in BLISS-52 and BLISS-76 had mucocutaneous (58.8%) and/or musculoskeletal (43.6%) manifestations at baseline, which have been shown to be associated with diminutions in physical HRQoL aspects (35, 36) and may therefore partially explain the observed high frequencies of adverse HRQoL outcomes in physical functioning and general health.

As expected, we observed increasing proportions of patients reporting adverse HRQoL outcomes and severe fatigue with increasing age, especially in physical outcomes, with more than 40% of SRI-4 responders aged 55+ years reporting adverse physical functioning and general health. The association between increasing age and adverse HRQoL outcomes was independent of other factors in multivariable logistic regression models, in line with the known negative impact of age on SLE patient's HRQoL (37). Interestingly, anti-dsDNA positivity at baseline was negatively associated with adverse outcome in physical HRQoL aspects and fatigue, as was anti-Sm positivity in mental HRQoL aspects and fatigue. Given the fact that more patients among responders had received belimumab rather than placebo (14, 15), this finding is in line with what is known about serological activity at baseline portending favourable response to belimumab therapy in clinical (38–40) and HRQoL (41) facets.

Established organ damage (accrued from disease onset until the time of evaluation, i.e., at week 52) in the musculoskeletal, mucocutaneous as well as cardiovascular and peripheral vascular SDI domains was associated with adverse HRQoL outcomes in physical but not mental aspects. By contrast, neuropsychiatric damage was associated with adverse outcome to treatment with regard to social functioning, mental health and fatigue. The latter finding, albeit not reaching statistical significance after adjustment, may provide support for investigation of functional or structural changes in the brain as potential contributors to the prominent fatigue in patients with SLE rather than or along with neuroinflammation, as also implicated in multiple sclerosis (42). Notably, we found no association between the degree of SLE disease activity and adverse HRQoL outcomes. Data in previous literature are conflicting regarding the effect of disease activity and organ damage on HRQoL, with some studies implicating a negative impact (43–45) and others reporting no evident connexion (37, 46, 47). These discrepancies could be partly explained by the different instruments used to measure patients' HRQoL, e.g., generic vs. disease-specific tools, or differences in clinical phenotypes. For instance, severe active lupus nephritis and neuropsychiatric SLE were excluded from the BLISS-52 and BLISS-76 trials, which disallows generalisability of our findings to these subgroups. Additionally, our definitions were applied to SRI-4 responders, i.e., patients who had attained a lower degree of activity relative to non-responders, which may constitute one of the reasons underlying the lack of association between disease activity and patient-reported HRQoL outcomes. Nevertheless, our data suggest that HRQoL outcomes are not solely dependent on clinical and serological features of disease activity, advocating use of patient-reported HRQoL as an integral part of the clinical assessment, as per current recommendations (48).

Patients of Black/African American and White/Caucasian ancestry reported the highest frequencies of adverse outcomes in most HRQoL domains. In logistic regression analysis, White/Caucasian ancestry was associated with adverse role physical, mental health and vitality using SF-36 and with severe fatigue using FACIT-F, independently of disease activity, organ damage and add-on belimumab. Conversely, Asian ancestry was associated with lower frequencies of adverse vitality and severe fatigue. Additionally, Hispanic/Latin American ethnicity was associated with lower frequencies of adverse physical functioning, adverse general health, adverse vitality and severe fatigue. Our findings are in line with observations from different multi-ethnic cohorts showing that patients of White/Caucasian ancestry generally report worse HRQoL than non-White/Caucasians (32), Hispanics (33) or Asians (49), despite a high variability across the studies in terms of study population, clinical features of the participants and selection of comparators. However, in a study by Kiani et al., White/Caucasians reported higher scores in SF-36 physical functioning and role emotional but lower scores in SF-36 vitality than Black/African Americans (46). One explanation for the discrepancies across ancestries and ethnic groups is likely traced to known differences regarding disease prevalence, clinical manifestations, disease activity and acquisition of organ damage (3, 4). Importantly, the determinants of HRQoL are multifactorial, and geographical, economical and sociocultural aspects are also expected to exert considerable influence on patients' HRQoL perception.

Data from both clinical trial and real-life settings suggest that the use of belimumab improves HRQoL along with improvements in disease activity, reduction of glucocorticoid doses, and prevention of severe flares (5, 6, 8, 9). In the present investigation comprising only patients who showed an adequate response to treatment, addition of belimumab 10 mg/kg to standard therapy was associated with lower proportions of adverse physical functioning and severe fatigue compared with standard therapy alone, independently of disease activity and organ damage at the time of the final evaluation. In the SWEFOT trial that compared addition of infliximab with addition of sulfasalazine and hydroxychloroquine in methotrexate-refractory early RA patients, no difference was found regarding proportions of patients achieving a good secondary response to treatment, but patients receiving infliximab reported less cumulative pain and less refractory pain after 21 months on the second-line therapy (50, 51). Although the administration route and, consequently, the visit frequency to the care unit may have exerted a placebo effect in favour of infliximab in SWEFOT, the preventive effects of biological agents against adverse patient-reported outcomes seen in both studies are supportive of molecular trajectories underlying these observations, and provide rationale for further investigation of the effects of biological therapies on HRQoL outcomes.

The *post-hoc* nature of our analysis constituted a major limitation. Furthermore, no data existed regarding epidemiological characteristics and comorbid conditions with a known impact on HRQoL, such as socioeconomical status, social relationships and co-existence of fibromyalgia or depression. We also lacked information about illness perceptions, which are known to impact on HRQoL (52, 53). Finally, disease-specific instruments for assessing HRQoL were not utilised in the BLISS trials; this likely underestimated HRQoL aspects of particular relevance for SLE populations. The aforementioned limitations together with the selected population of the BLISS trials may collectively weaken the external validity of our findings. Nonetheless, strengths of this investigation included the large study population, participation of patients from 32 different countries which allowed us to compare patients of different ancestries and ethnic origins, and the high degree of completeness of the data provided from the CSDR consortium which conferred power on statistical analyses and allowed inclusion of multiple factors in regression models.

In summary, substantial proportions of SLE patients reported adverse HRQoL outcomes despite a documented clinical improvement after a 52-week long therapy, especially in physical aspects. Particularly high proportions were seen within Black/African American and White/Caucasian patients. Notably, addition of belimumab 10 mg/kg to standard therapy exerted a preventive effect against adverse physical functioning and severe fatigue. Our results corroborate that HRQoL diminutions constitute a substantial burden in patients with SLE, and highlight the limitations of current therapeutic strategies. Further investigation of underlying factors is merited, for instance, identification of potential explanations underlying the impact of musculoskeletal, mucocutaneous and cardiovascular damage, toward the development of personalised interventions aiming at improving HRQoL outcomes.

## Data Availability Statement

The raw data supporting the conclusions of this article will be made available by the authors, without undue reservation.

## Ethics Statement

The studies involving human participants were reviewed and approved by Swedish Ethical Review Authority (2019-05498). The patients/participants provided their written informed consent to participate in this study.

## Author Contributions

AG, VQ, AC, YE, JLa, and IP: study conception and design. AG, VQ, AC, AB, JLi, and IP: acquisition of data. AG, VQ, AC, SE, YE, JLa, and IP: analysis and interpretation of data. All authors were involved in the drafting of the manuscript or revising it critically for important intellectual content, and all authors approved the final version to be submitted for publication.

## Conflict of Interest

IP has received research funding and/or honoraria from Amgen, Elli Lilly and Company, Gilead Sciences, GlaxoSmithKline and Novartis. The remaining authors declare that the research was conducted in the absence of any commercial or financial relationships that could be construed as a potential conflict of interest.
