# Supplementary material for: Adverse Health-Related Quality of Life Outcome Despite Adequate Clinical Response to Treatment in Systemic Lupus Erythematosus
Source: Front Med (Lausanne). 2021 Apr 16;8:651249. doi: 10.3389/fmed.2021.651249 (PMC8085308; doi:10.3389/fmed.2021.651249)
Supplement: Supplementary file 1 [file Table_1.DOCX]

| **Country group** | **Country** | **Number of patients** |
| --- | --- | --- |
| **Asia Pacific**  (N = 143) | 🇦🇺 Australia | 5 |
|  | 🇭🇰 Hong Kong | 2 |
|  | 🇮🇳 India | 35 |
|  | 🇰🇷 Korea | 24 |
|  | 🇵🇭 Philippines | 40 |
|  | 🇹🇼 Taiwan | 37 |
| **Canada/USA**  (N = 151) | 🇨🇦 Canada | 3 |
|  | 🇺🇸 USA | 148 |
| **Europe/Israel**  (N = 176) | 🇦🇹 Austria | 12 |
|  | 🇧🇪 Belgium | 4 |
|  | 🇨🇿 Czech Republic | 17 |
|  | 🇫🇷 France | 7 |
|  | 🇩🇪 Germany | 31 |
|  | 🇮🇱 Israel | 10 |
|  | 🇮🇹 Italy | 8 |
|  | 🇳🇱 Netherlands | 3 |
|  | 🇵🇱 Poland | 24 |
|  | 🇷🇴 Romania | 18 |
|  | 🇷🇺 Russia | 39 |
|  | 🇸🇪 Sweden | 1 |
|  | 🇬🇧 United Kingdom | 2 |
| **Latin America**  (N = 290) | 🇦🇷 Argentina | 51 |
|  | 🇧🇷 Brazil | 45 |
|  | 🇨🇱 Chile | 16 |
|  | 🇨🇴 Colombia | 100 |
|  | 🇨🇷 Costa Rica | 3 |
|  | 🇲🇽 Mexico | 41 |
|  | 🇵🇪 Peru | 29 |
|  | 🇵🇷 Puerto Rico | 5 |

**Supplementary Table 1.** Numbers of SRI-4 responders in the pooled BLISS study population across countries of residence and country groups.

The table shows the number of patients who met the primary endpoint of the BLISS-52 and BLISS-76 clinical trials across countries of residence. Countries were classified into four categories according to their geographical location.

N, total number of patients; SRI-4, SLE Responder Index 4.
