# Supplementary material for: Adverse Health-Related Quality of Life Outcome Despite Adequate Clinical Response to Treatment in Systemic Lupus Erythematosus
Source: Front Med (Lausanne). 2021 Apr 16;8:651249. doi: 10.3389/fmed.2021.651249 (PMC8085308; doi:10.3389/fmed.2021.651249)
Supplement: Supplementary file 2 [file Table_2.DOCX]

**Supplementary Table 2.** Comparisons of SF-36 scale scores between SRI-4 responders and matched population-based norms.

|  | SRI-4  non-responders  N = 760 | | SRI-4  responders  N = 760 | General population  N = 760 | P value | x MCID |
| --- | --- | --- | --- | --- | --- | --- |
| SF-36 items |  |  | | | | |
| PCS | 41.3 ± 10.3 | | 44.5 ± 9.0 | 51.1 ± 2.4 | **< 0.001** | 2.6 |
| MCS | 42.7 ± 12.1 | | 44.9 ± 10.7 | 48.9 ± 1.0 | **< 0.001** | 1.6 |
| PF | 63.6 ± 27.1 | | 69.3 ± 23.5 | 86.6 ± 5.2 | **< 0.001** | 3.5 |
| RP | 58.4 ± 28.5 | | 65.1 ± 24.0 | 83.3 ± 5.0 | **< 0.001** | 3.6 |
| BP | 55.6 ± 26.9 | | 63.8 ± 23.5 | 76.3 ± 4.5 | **< 0.001** | 2.5 |
| GH | 44.9 ± 21.3 | | 52.4 ± 20.4 | 73.2 ± 3.7 | **< 0.001** | 4.2 |
| VT | 48.7 ± 24.1 | | 55.5 ± 20.9 | 59.4 ± 1.6 | **< 0.001** | 0.8 |
| SF | 63.3 ± 27.3 | | 70.3 ± 24.1 | 83.0 ± 1.6 | **< 0.001** | 2.5 |
| RE | 65.0 ± 28.0 | | 69.8 ± 24.0 | 81.0 ± 1.8 | **< 0.001** | 2.2 |
| MH | 63.7 ± 21.2 | | 67.1 ± 19.3 | 73.2 ± 1.2 | **< 0.001** | 1.2 |

Data are presented as means ± standard deviation SF-36 scale scores reported by patients with SLE who achieved an adequate clinical response following a 52-week long intervention with standard therapy plus belimumab or placebo compared with US population-based age- and sex-matched norms. P values are derived from non-parametric Wilcoxon signed rank tests for paired samples. Statistically significant P values are in bold. The last column displays the corresponding number of times the difference in scores exceeding the MCID, i.e. 2.5 points for SF-36 component summary scores and 5.0 points for subscale scores.

BP, bodily pain; GH, general health; MCID, minimal clinically important difference; MCS, mental component summary; MH, mental health; PCS, physical component summary; PF, physical functioning; RE, role emotional; RP, role physical; SF, social functioning; SF-36, short form 36; SLE, systemic lupus erythematosus; SRI-4, SLE Responder Index 4; VT, vitality.
