# Supplementary material for: Adverse Health-Related Quality of Life Outcome Despite Adequate Clinical Response to Treatment in Systemic Lupus Erythematosus
Source: Front Med (Lausanne). 2021 Apr 16;8:651249. doi: 10.3389/fmed.2021.651249 (PMC8085308; doi:10.3389/fmed.2021.651249)
Supplement: Supplementary file 4 [file Table_4.DOCX]

**Supplementary Table 4.** Frequencies of adverse HRQoL outcomes in SRI-4 responders at baseline and week 52.

| **HRQoL outcome** | **SRI-4 responders at baseline** | | **SRI-4 responders at week 52** | | **P value** |
| --- | --- | --- | --- | --- | --- |
|  | **n** | **%** | **n** | **%** |  |
| **SF-36 items** | **N = 760** | | **N = 760** | |  |
| PCS ≤ NP5 | 274 | 36.5 | 132 | 17.4 | **< 0.001** |
| MCS ≤ NP5 | 222 | 29.6 | 126 | 16.6 | **< 0.001** |
| PF ≤ NP5 | 304 | 40.2 | 193 | 25.4 | **< 0.001** |
| RP ≤ NP5 | 137 | 18.1 | 57 | 7.5 | **< 0.001** |
| BP ≤ NP5 | 216 | 28.5 | 94 | 12.4 | **< 0.001** |
| GH ≤ NP5 | 376 | 49.9 | 221 | 29.1 | **< 0.001** |
| VT ≤ NP5 | 165 | 21.8 | 81 | 10.7 | **< 0.001** |
| SF ≤ NP5 | 198 | 26.1 | 99 | 13.0 | **< 0.001** |
| RE ≤ NP5 | 92 | 12.2 | 50 | 6.6 | **< 0.001** |
| MH ≤ NP5 | 154 | 20.4 | 75 | 9.9 | **< 0.001** |
| **FACIT-F** | **N = 742** | | **N = 745** | |  |
| FACIT-F < 30 | 317 | 42.7 | 192 | 25.8 | **< 0.001** |

Data are presented as numbers (n) and proportions (%) of SRI-4 responders who reported adverse HRQoL outcomes at baseline and week 52 from treatment initiation. P values are derived from Wilcoxon signed rank tests. Statistically significant P values are in bold.

BP, bodily pain; FACIT-F, Functional Assessment of Chronic Illness Therapy - Fatigue; GH, general health; HRQoL, health-related quality of life; MCS, mental component summary; MH, mental health; NP5, normative 5^th^ percentile; PCS, physical component summary; PF, physical functioning; RE, role emotional; RP, role physical; SF, social functioning; SF-36, short form-36; SRI-4, SLE responder index 4 ; VT, vitality.
