# Supplementary material for: Adverse Health-Related Quality of Life Outcome Despite Adequate Clinical Response to Treatment in Systemic Lupus Erythematosus
Source: Front Med (Lausanne). 2021 Apr 16;8:651249. doi: 10.3389/fmed.2021.651249 (PMC8085308; doi:10.3389/fmed.2021.651249)
Supplement: Supplementary file 5 [file Table_5.DOCX]

**Supplementary Table 5.** Demographics and clinical characteristics of SRI-4 responders reporting adverse and non-adverse PCS at week 52 in the pooled BLISS study population.

|  | SRI-4 responders  N = 760 | Adverse PCS  N = 132 | Non-adverse PCS  N = 628 | P value |
| --- | --- | --- | --- | --- |
| Patient characteristics | | | |  |
| Age at baseline (years) | 37.3 ± 11.4 | 41.1 ± 11.8 | 36.5 ± 11.1 | **<0.001** |
| Female sex | 717 (94.3%) | 125 (94.7%) | 592 (94.3%) | 0.846 |
| Ancestries |  |  |  |  |
| Asian | 144 (18.9%) | 18 (13.6%) | 126 (20.1%) | 0.087 |
| Black/African American | 55 (7.2%) | 12 (9.1%) | 43 (6.8%) | 0.366 |
| Indigenous American* | 210 (27.6%) | 27 (20.5%) | 183 (29.1%) | **0.043** |
| White/Caucasian | 351 (46.2%) | 75 (56.8%) | 276 (43.9%) | **0.007** |
| Clinical data | | | |  |
| SLE duration at baseline (years) | 5.8 (1.2−8.5) | 4.2 (1.1−10.1) | 3.8 (1.2−8.0) | 0.272 |
| SLEDAI-2K score |  |  |  |  |
| Baseline | 10.7 ± 3.6 | 10.7 ± 4.0 | 10.7 ± 3.5 | 0.664 |
| Week 52 | 3.8 ± 2.9 | 3.5 ± 3.2 | 3.9 ± 2.8 | 0.128 |
| SDI score |  |  |  |  |
| Baseline | 0.7 ± 1.1  0.0 (0.0−1.0) | 1.1 ± 1.4  1.0 (0.0−1.8) | 0.6 ± 1.1  0.0 (0.0−1.0) | **<0.001** |
| Week 52 | 0.7 ± 1.2  0.0 (0.0−1.0) | 1.1 ± 1.4  1.0 (0.0−2.0) | 0.6 ± 1.1  0.0 (0.0−1.0) | **<0.001** |
| SDI score > 0 |  |  |  |  |
| Baseline | 293 (38.6%) | 72 (54.5%) | 221 (35.2%) | **<0.001** |
| Week 52 | 307 (40.4%) | 77 (58.3%) | 230 (36.6%) | **<0.001** |
| Serological profile at baseline |  |  |  |  |
| Anti-dsDNA (+) | 517 (68.0%) | 73 (55.3%) | 444 (70.7%) | **0.001** |
| Anti-Sm (+) | 224 (29.6%); N = 758 | 31 (23.5%) | 193 (30.8%); N = 626 | 0.093 |
| Low C3 | 311 (40.9%) | 37 (28.0%) | 274 (43.6%) | **0.001** |
| Low C4 | 395 (52.0%) | 51 (38.6%) | 344 (54.8%) | **0.001** |
| Prednisone eq. dose (mg/day) |  |  |  |  |
| Baseline | 11.7 ± 9.0 | 9.7 ± 7.6 | 12.1 ± 9.3 | **0.017** |
| Week 52 | 8.7 ± 6.8; N = 754 | 8.1 ± 6.3; N = 131 | 8.8 ± 7.0; N = 623 | 0.525 |
| Antimalarial agents at week 52^†^ | 478 (62.9%) | 76 (57.6%) | 402 (64.0%) | 0.164 |
| Immunosuppressants at week 52 |  |  |  |  |
| Azathioprine | 149 (19.6%) | 24 (18.2%) | 125 (19.9%) | 0.650 |
| Methotrexate | 78 (10.3%) | 23 (17.4%) | 55 (8.8%) | **0.003** |
| Mycophenolic acid | 72 (9.5%) | 9 (6.8%) | 63 (10.0%) | 0.252 |
| Other immunosuppressants^‡^ | 15 (2.0%) | 5 (3.8%) | 10 (1.6%) | 0.099 |
| Trial intervention |  |  |  |  |
| Placebo | 217 (28.6%) | 39 (29.5%) | 178 (28.3%) | 0.781 |
| Belimumab 1 mg/kg | 258 (33.9%) | 48 (36.4%) | 210 (33.4%) | 0.519 |
| Belimumab 10 mg/kg | 285 (37.5%) | 45 (34.1%) | 240 (38.2%) | 0.373 |

Data are presented as numbers (percentage) or means ± standard deviation. In case of non-normal distributions, medians (interquartile range) are indicated. In case of missing values, the total number of patients with available data is indicated. Statistically significant P values are in bold.

* Alaska Native or American Indian from North, South or Central America.

^†^ Hydroxychloroquine, chloroquine, mepacrine, mepacrine hydrochloride or quinine sulfate.

^‡^ Cyclosporine, oral cyclophosphamide, leflunomide, mizoribine or thalidomide.

C3 = complement component protein 3; C4 = complement component protein 4; dsDNA = double stranded DNA; SDI = Systemic Lupus International Collaborating Clinics (SLICC)/American College of Rheumatology (ACR) Damage Index; SLE = systemic lupus erythematosus; SLEDAI-2K = SLE Disease Activity Index 2000; Sm = Smith; SRI-4 = SLE Responder Index 4.
