# Supplementary material for: Adverse Health-Related Quality of Life Outcome Despite Adequate Clinical Response to Treatment in Systemic Lupus Erythematosus
Source: Front Med (Lausanne). 2021 Apr 16;8:651249. doi: 10.3389/fmed.2021.651249 (PMC8085308; doi:10.3389/fmed.2021.651249)
Supplement: Supplementary file 6 [file Table_6.DOCX]

**Supplementary Table 6.** Demographics and clinical characteristics of SRI-4 responders reporting adverse and non-adverse MCS at week 52 in the pooled BLISS study population

|  | SRI-4 responders  N = 760 | Adverse MCS  N = 126 | Non-adverse MCS  N = 634 | P value |
| --- | --- | --- | --- | --- |
| Patient characteristics | | | |  |
| Age at baseline (years) | 37.3 ± 11.4 | 38.6 ± 11.1 | 37.0 ± 11.4 | 0.093 |
| Female sex | 717 (94.3%) | 116 (92.1%) | 601 (94.8%) | 0.225 |
| Ancestries |  |  |  |  |
| Asian | 144 (18.9%) | 22 (17.5%) | 122 (19.2%) | 0.641 |
| Black/African American | 55 (7.2%) | 6 (4.8%) | 49 (7.7%) | 0.240 |
| Indigenous American* | 210 (27.6%) | 23 (18.3%) | 187 (29.5%) | **0.010** |
| White/Caucasian | 351 (46.2%) | 75 (59.5%) | 276 (43.5%) | **0.001** |
| Clinical data | | | |  |
| SLE duration at baseline (years) | 5.8 (1.2−8.5) | 4.4 (1.5−8.1) | 3.9 (1.1−8.8) | 0.678 |
| SLEDAI-2K score |  |  |  |  |
| Baseline | 10.7 ± 3.6 | 10.6 ± 3.7 | 10.7 ± 3.6 | 0.713 |
| Week 52 | 3.8 ± 2.9 | 4.0 ± 3.0 | 3.8 ± 2.9 | 0.594 |
| SDI score |  |  |  |  |
| Baseline | 0.7 ± 1.1  0.0 (0.0−1.0) | 0.7 ± 1.4  0.0 (0.0−1.0) | 0.7 ± 1.1  0.0 (0.0−1.0) | 0.778 |
| Week 52 | 0.7 ± 1.2  0.0 (0.0−1.0) | 0.7 ± 1.4  0.0 (0.0−1.0) | 0.7 ± 1.1  0.0 (0.0−1.0) | 0.986 |
| SDI score > 0 |  |  |  |  |
| Baseline | 293 (38.6%) | 48 (38.1%) | 245 (38.6%) | 0.908 |
| Week 52 | 307 (40.4%) | 52 (41.3%) | 255 (40.2%) | 0.827 |
| Serological profile at baseline |  |  |  |  |
| Anti-dsDNA (+) | 517 (68.0%) | 82 (65.1%) | 435 (68.6%) | 0.437 |
| Anti-Sm (+) | 224 (29.6%); N = 758 | 25 (19.8%) | 199 (31.5%); N = 632 | **0.009** |
| Low C3 | 311 (40.9%) | 49 (38.9%) | 262 (41.3%) | 0.611 |
| Low C4 | 395 (52.0%) | 65 (51.6%) | 330 (52.1%) | 0.924 |
| Prednisone eq. dose (mg/day) |  |  |  |  |
| Baseline | 11.7 ± 9.0 | 11.9 ± 10.5 | 11.6 ± 8.7 | 0.396 |
| Week 52 | 8.7 ± 6.8; N = 754 | 9.2 ± 7.5 | 8.6 ± 6.7; N = 628 | 0.625 |
| Antimalarial agents at week 52^†^ | 478 (62.9%) | 77 (61.1%) | 401 (63.2%) | 0.650 |
| Immunosuppressants at week 52 |  |  |  |  |
| Azathioprine | 149 (19.6%) | 19 (12.1%) | 130 (20.5%) | 0.161 |
| Methotrexate | 78 (10.3%) | 16 (12.7%) | 62 (9.8%) | 0.324 |
| Mycophenolic acid | 72 (9.5%) | 16 (12.7%) | 56 (8.8%) | 0.176 |
| Other immunosuppressants^‡^ | 15 (2.0%) | 1 (0.8%) | 14 (2.2%) | 0.487 |
| Trial intervention |  |  |  |  |
| Placebo | 217 (28.6%) | 39 (31.0%) | 178 (28.1%) | 0.781 |
| Belimumab 1 mg/kg | 258 (33.9%) | 38 (30.2%) | 220 (34.7%) | 0.325 |
| Belimumab 10 mg/kg | 285 (37.5%) | 49 (38.9%) | 236 (37.2%) | 0.724 |

Data are presented as numbers (percentage) or means ± standard deviation. In case of non-normal distributions, medians (interquartile range) are indicated. In case of missing values, the total number of patients with available data is indicated. Statistically significant P values are in bold.

* Alaska Native or American Indian from North, South or Central America.

^†^ Hydroxychloroquine, chloroquine, mepacrine, mepacrine hydrochloride or quinine sulfate.

^‡^ Cyclosporine, oral cyclophosphamide, leflunomide, mizoribine or thalidomide.

C3 = complement component protein 3; C4 = complement component protein 4; dsDNA = double stranded DNA; SDI = Systemic Lupus International Collaborating Clinics (SLICC)/American College of Rheumatology (ACR) Damage Index; SLE = systemic lupus erythematosus; SLEDAI-2K = SLE Disease Activity Index 2000; Sm = Smith; SRI-4 = SLE Responder Index 4.
