# Supplementary material for: Adverse Health-Related Quality of Life Outcome Despite Adequate Clinical Response to Treatment in Systemic Lupus Erythematosus
Source: Front Med (Lausanne). 2021 Apr 16;8:651249. doi: 10.3389/fmed.2021.651249 (PMC8085308; doi:10.3389/fmed.2021.651249)
Supplement: Supplementary file 7 [file Table_7.DOCX]

**Supplementary Table 7.** Demographics and clinical characteristics of SRI-4 responders reporting severe and non-severe FACIT-F at week 52 in the pooled BLISS study population

|  | SRI-4 responders  N = 760 | FACIT-F <30  N = 192 | FACIT-F ≥30  N = 553 | P value |
| --- | --- | --- | --- | --- |
| Patient characteristics | | | |  |
| Age at baseline (years) | 37.3 ± 11.4 | 41.0 ± 11.1 | 36.1 ± 11.3 | **<0.001** |
| Female sex | 717 (94.3%) | 181 (94.3%) | 521 (94.2%) | 0.977 |
| Ancestries |  |  |  |  |
| Asian | 144 (18.9%) | 28 (14.6%) | 101 (18.3%) | 0.246 |
| Black/African American | 55 (7.2%) | 19 (9.9%) | 36 (6.5%) | 0.122 |
| Indigenous American* | 210 (27.6%) | 30 (15.6%) | 180 (32.5%) | **<0.001** |
| White/Caucasian | 351 (46.2%) | 115 (59.9%) | 236 (42.7%) | **<0.001** |
| Clinical data | | | |  |
| SLE duration at baseline (years) | 5.8 (1.2−8.5) | 5.1 (1.0−7.8) | 3.9 (1.1−8.5) | 0.728 |
| SLEDAI-2K score |  |  |  |  |
| Baseline | 10.7 ± 3.6 | 10.4 ± 3.4 | 10.7 ± 3.7 | 0.303 |
| Week 52 | 3.8 ± 2.9 | 3.7 ± 3.0 | 3.8 ± 2.8 | 0.424 |
| SDI score |  |  |  |  |
| Baseline | 0.7 ± 1.1  0.0 (0.0−1.0) | 0.8 ± 1.3  0.0 (0.0−1.0) | 0.6 ± 1.1  0.0 (0.0−1.0) | **0.028** |
| Week 52 | 0.7 ± 1.2  0.0 (0.0−1.0) | 0.9 ± 1.4  0.0 (0.0−1.0) | 0.7 ± 1.1  0.0 (0.0−1.0) | **0.016** |
| SDI score > 0 |  |  |  |  |
| Baseline | 293 (38.6%) | 88 (45.8%) | 204 (36.9%) | **0.029** |
| Week 52 | 307 (40.4%) | 93 (48.4%) | 213 (38.5%) | **0.016** |
| Serological profile at baseline |  |  |  |  |
| Anti-dsDNA (+) | 517 (68.0%) | 111 (57.8%) | 395 (71.4%) | **<0.001** |
| Anti-Sm (+) | 224 (29.6%); N = 758 | 40 (20.8%) | 180 (32.7%) | **0.002** |
| Low C3 | 311 (40.9%) | 65 (33.9%) | 237 (42.9%) | **0.029** |
| Low C4 | 395 (52.0%) | 84 (43.8%) | 303 (54.8%) | **0.008** |
| Prednisone eq. dose (mg/day) |  |  |  |  |
| Baseline | 11.7 ± 9.0 | 9.7 ± 8.9 | 12.2 ± 8.9 | **<0.001** |
| Week 52 | 8.7 ± 6.8; N = 754 | 7.9 ± 7.0; N = 191 | 9.0 ± 6.8; N = 548 | **0.008** |
| Antimalarial agents at week 52^†^ | 478 (62.9%) | 115 (59.9%) | 358 (64.7%) | 0.230 |
| Immunosuppressants at week 52 |  |  |  |  |
| Azathioprine | 149 (19.6%) | 33 (17.2%) | 116 (21.0%) | 0.258 |
| Methotrexate | 78 (10.3%) | 28 (14.6%) | 50 (9.0%) | **0.031** |
| Mycophenolic acid | 72 (9.5%) | 22 (11.5%) | 50 (9.0%) | 0.329 |
| Other immunosuppressants^‡^ | 15 (2.0%) | 4 (2.1%) | 11 (2.0%) | 0.936 |
| Trial intervention |  |  |  |  |
| Placebo | 217 (28.6%) | 66 (34.4%) | 148 (26.8%) | **0.045** |
| Belimumab 1 mg/kg | 258 (33.9%) | 70 (36.5%) | 185 (33.5%) | 0.450 |
| Belimumab 10 mg/kg | 285 (37.5%) | 56 (29.2%) | 220 (39.8%) | **0.009** |

Data are presented as numbers (percentage) or means ± standard deviation. In case of non-normal distributions, medians (interquartile range) are indicated. In case of missing values, the total number of patients with available data is indicated. Statistically significant P values are in bold.

* Alaska Native or American Indian from North, South or Central America.

^†^ Hydroxychloroquine, chloroquine, mepacrine, mepacrine hydrochloride or quinine sulfate.

^‡^ Cyclosporine, oral cyclophosphamide, leflunomide, mizoribine or thalidomide.

C3 = complement component protein 3; C4 = complement component protein 4; dsDNA = double stranded DNA; SDI = Systemic Lupus International Collaborating Clinics (SLICC)/American College of Rheumatology (ACR) Damage Index; SLE = systemic lupus erythematosus; SLEDAI-2K = SLE Disease Activity Index 2000; Sm = Smith; SRI-4 = SLE Responder Index 4.
