# Supplementary material for: Adverse Health-Related Quality of Life Outcome Despite Adequate Clinical Response to Treatment in Systemic Lupus Erythematosus
Source: Front Med (Lausanne). 2021 Apr 16;8:651249. doi: 10.3389/fmed.2021.651249 (PMC8085308; doi:10.3389/fmed.2021.651249)
Supplement: Supplementary file 8 [file Table_8.DOCX]

**Supplementary Table 8.** Associations between SDI domains and adverse PCS, MCS and FACIT-F.

| **SDI domain** | | **PCS** | | | **MCS** | | | **FACIT-F** | | |
| --- | --- | --- | --- | --- | --- | --- | --- | --- | --- | --- |
|  |  | **OR** | **95% CI** | **P value** | **OR** | **95% CI** | **P value** | **OR** | **95% CI** | **P value** |
| **Ocular** | **Unadj.** | 0.72 | 0.33–1.56 | 0.400 | 1.00 | 0.50–2.02 | 0.993 | 0.76 | 0.40–1.46 | 0.409 |
|  | **Adj.** | 0.47 | 0.21–1.07 | 0.074 | 0.85 | 0.41–1.74 | 0.654 | 0.45 | 0.23–0.91 | **0.027** |
| **Neuropsychiatric** | **Unadj.** | 1.69 | 1.15–2.49 | **0.008** | 1.26 | 0.82–1.92 | 0.295 | 1.50 | 1.04–2.16 | **0.031** |
|  | **Adj.** | 1.39 | 0.92–2.09 | 0.115 | 1.10 | 0.70–1.72 | 0.671 | 1.12 | 0.76–1.65 | 0.573 |
| **Renal** | **Unadj.** | 1.75 | 0.55–5.57 | 0.346 | 2.57 | 0.86–7.65 | 0.090 | 1.04 | 0.33–3.32 | 0.941 |
|  | **Adj.** | 1.79 | 0.53–6.11 | 0.350 | 2.56 | 0.82–7.98 | 0.105 | 1.00 | 0.29–3.49 | 0.999 |
| **Pulmonary** | **Unadj.** | 1.29 | 0.60–2.80 | 0.516 | 0.93 | 0.37–2.35 | 0.884 | 1.20 | 0.59–2.44 | 0.623 |
|  | **Adj.** | 1.20 | 0.55–2.64 | 0.648 | 0.85 | 0.32–2.27 | 0.747 | 1.12 | 0.53–2.35 | 0.770 |
| **Cardiovascular** | **Unadj.** | 2.75 | 1.42–5.33 | **0.003** | 1.16 | 0.52–2.58 | 0.716 | 1.70 | 0.89–3.24 | 0.111 |
|  | **Adj.** | 2.12 | 1.07–4.21 | **0.032** | 0.95 | 0.42–2.16 | 0.904 | 1.16 | 0.58–2.31 | 0.682 |
| **Peripheral vascular** | **Unadj.** | 1.64 | 0.92–2.91 | 0.095 | 0.47 | 0.15–1.42 | 0.180 | 1.24 | 0.70–2.18 | 0.461 |
|  | **Adj.** | 1.52 | 0.83–2.77 | 0.174 | 0.43 | 0.14–1.35 | 0.149 | 1.09 | 0.59–2.01 | 0.787 |
| **Gastrointestinal** | **Unadj.** | 2.12 | 1.07–4.22 | **0.032** | 0.91 | 0.38–2.18 | 0.836 | 2.01 | 1.05–3.82 | **0.034** |
|  | **Adj.** | 1.80 | 0.88–3.67 | 0.106 | 0.77 | 0.32–1.88 | 0.568 | 1.57 | 0.79–3.10 | 0.197 |
| **Musculoskeletal** | **Unadj.** | 1.61 | 1.17–2.21 | **0.003** | 0.80 | 0.52–1.24 | 0.319 | 1.10 | 0.80–1.51 | 0.545 |
|  | **Adj.** | 1.41 | 1.01–1.96 | **0.041** | 0.74 | 0.47–1.16 | 0.184 | 0.90 | 0.64–1.26 | 0.540 |
| **Skin** | **Unadj.** | 1.68 | 0.95–2.95 | 0.072 | 1.23 | 0.66–2.29 | 0.524 | 1.07 | 0.61–1.88 | 0.818 |
|  | **Adj.** | 1.44 | 0.80–2.61 | 0.228 | 1.12 | 0.58–2.14 | 0.740 | 0.80 | 0.43–1.46 | 0.459 |
| **Gonadal failure** | **Unadj.** | 1.00 | 1.00–1.00 | 0.703 | 1.00 | 1.00–1.00 | 0.706 | 1.00 | 1.00–1.00 | 0.663 |
|  | **Adj.** | 1.00 | 0.97–1.00 | 0.782 | 1.00 | 1.00–1.00 | 0.744 | 1.00 | 1.00–1.00 | 0.691 |
| **Diabetes** | **Unadj.** | 1.00 | 1.00–1.00 | 0.702 | 1.00 | 1.00–1.00 | 0.707 | 1.00 | 1.00–1.00 | 0.663 |
|  | **Adj.** | 1.00 | 1.00–1.00 | 0.779 | 1.00 | 1.00–1.00 | 0.744 | 1.00 | 1.00–1.00 | 0.691 |
| **Malignancy** | **Unadj.** | 1.00 | 1.00–1.00 | 0.703 | 1.00 | 1.00–1.00 | 0.708 | 1.00 | 1.00–1.00 | 0.664 |
|  | **Adj.** | 1.00 | 1.00–1.00 | 0.782 | 1.00 | 1.00–1.00 | 0.745 | 1.00 | 1.00–1.00 | 0.691 |

Data are presented as unadjusted (upper row) and adjusted (lower row) OR, 95% CI and P value deriving from logistic regression analyses. Covariates in multivariable logistic regression models included age, sex, ancestry, Hispanic ethnicity, SLEDAI-2K and SDI scores at week 52 and the trial intervention. Statistically significant P values are in bold.

Adj., Adjusted; CI, confidence interval; MCS, mental component summary; OR, odds ratio; PCS, physical component summary; Unadj., unadjusted.
