# Supplementary material for: Adverse Health-Related Quality of Life Outcome Despite Adequate Clinical Response to Treatment in Systemic Lupus Erythematosus
Source: Front Med (Lausanne). 2021 Apr 16;8:651249. doi: 10.3389/fmed.2021.651249 (PMC8085308; doi:10.3389/fmed.2021.651249)
Supplement: Supplementary file 9 [file Table_9.DOCX]

**Supplementary Table 9.** Associations between SDI domains and adverse SF-36 physical subscales.

| **SDI domain** | | **PF** | | | **RP** | | | **BP** | | | **GH** | | |
| --- | --- | --- | --- | --- | --- | --- | --- | --- | --- | --- | --- | --- | --- |
|  |  | **OR** | **95% CI** | **P value** | **OR** | **95% CI** | **P value** | **OR** | **95% CI** | **P value** | **OR** | **95% CI** | **P value** |
| **Ocular** | **Unadj.** | 1.02 | 0.56–1.86 | 0.939 | 0.48 | 0.12–1.96 | 0.308 | 0.73 | 0.30–1.80 | 0.494 | 0.94 | 0.52–1.68 | 0.827 |
|  | **Adj.** | 0.68 | 0.36–1.28 | 0.227 | 0.29 | 0.07–1.18 | 0.084 | 0.53 | 0.21–1.36 | 0.188 | 0.66 | 0.36–1.22 | 0.188 |
| **Neuropsychiatric** | **Unadj.** | 1.52 | 1.05–2.19 | **0.026** | 1.87 | 1.16–3.02 | **0.010** | 1.32 | 0.83–2.10 | 0.241 | 1.32 | 0.92–1.90 | 0.138 |
|  | **Adj.** | 1.24 | 0.84–1.83 | 0.285 | 1.47 | 0.88–2.46 | 0.144 | 1.10 | 0.67–1.79 | 0.712 | 1.05 | 0.72–1.54 | 0.789 |
| **Renal** | **Unadj.** | 2.62 | 0.94–7.32 | 0.066 | 0.90 | 0.11–6.79 | 0.899 | 1.80 | 0.50–6.47 | 0.374 | 0.88 | 0.28–2.80 | 0.830 |
|  | **Adj.** | 2.85 | 0.94–8.64 | 0.065 | 0.78 | 0.10–6.37 | 0.815 | 1.49 | 0.39–5.64 | 0.557 | 0.86 | 0.26–2.88 | 0.803 |
| **Pulmonary** | **Unadj.** | 1.76 | 0.89–3.48 | 0.105 | 1.76 | 0.73–4.22 | 0.210 | 0.66 | 0.18–2.44 | 0.536 | 1.36 | 0.69–2.68 | 0.376 |
|  | **Adj.** | 1.50 | 0.74–3.03 | 0.261 | 1.41 | 0.54–3.68 | 0.477 | 0.53 | 0.13–2.08 | 0.359 | 1.24 | 0.62–2.49 | 0.543 |
| **Cardiovascular** | **Unadj.** | 1.73 | 0.90–3.30 | 0.099 | 2.70 | 1.20–6.06 | **0.016** | 1.60 | 0.72–3.55 | 0.248 | 1.99 | 1.05–3.76 | **0.034** |
|  | **Adj.** | 1.32 | 0.66–2.62 | 0.434 | 1.99 | 0.84–4.72 | 0.118 | 1.26 | 0.55–2.89 | 0.584 | 1.51 | 0.79–2.92 | 0.216 |
| **Peripheral vascular** | **Unadj.** | 1.36 | 0.78–2.37 | 0.277 | 0.98 | 0.35–2.71 | 0.966 | 0.99 | 0.44–2.22 | 0.972 | 2.44 | 1.36–4.38 | **0.003** |
|  | **Adj.** | 1.27 | 0.71–2.27 | 0.430 | 0.87 | 0.30–2.56 | 0.799 | 0.93 | 0.41–2.12 | 0.859 | 2.32 | 1.28–4.17 | **0.005** |
| **Gastrointestinal** | **Unadj.** | 1.64 | 0.85–3.17 | 0.139 | 2.25 | 0.93–5.42 | 0.071 | 1.29 | 0.54–3.07 | 0.573 | 1.69 | 0.89–3.22 | 0.107 |
|  | **Adj.** | 1.31 | 0.65–2.63 | 0.449 | 1.82 | 0.72–4.62 | 0.207 | 1.05 | 0.43–2.58 | 0.917 | 1.41 | 0.73–2.73 | 0.312 |
| **Musculoskeletal** | **Unadj.** | 2.08 | 1.53–2.82 | **0.000** | 1.29 | 0.82–2.04 | 0.272 | 1.36 | 0.94–1.97 | 0.101 | 1.47 | 1.09–1.97 | **0.011** |
|  | **Adj.** | 1.80 | 1.31–2.47 | **0.000** | 1.03 | 0.63–1.68 | 0.910 | 1.23 | 0.84–1.80 | 0.287 | 1.29 | 0.95–1.75 | 0.104 |
| **Skin** | **Unadj.** | 2.42 | 1.44–4.06 | **0.001** | 0.73 | 0.24–2.23 | 0.583 | 1.67 | 0.89–3.12 | 0.111 | 1.25 | 0.74–2.12 | 0.405 |
|  | **Adj.** | 2.17 | 1.26–3.75 | **0.005** | 0.58 | 0.18–1.84 | 0.352 | 1.44 | 0.75–2.76 | 0.270 | 1.05 | 0.60–1.82 | 0.867 |
| **Gonadal failure** | **Unadj.** | 1.00 | 1.00–1.00 | 0.666 | 1.00 | 1.00–1.00 | 0.815 | 1.00 | 1.00–1.00 | 0.787 | 1.00 | 1.00–1.00 | 0.570 |
|  | **Adj.** | 1.00 | 1.00–1.00 | 0.671 | 1.00 | 0.99–1.01 | 0.887 | 1.00 | 1.00–1.00 | 0.807 | 1.00 | 1.00–1.00 | 0.673 |
| **Diabetes** | **Unadj.** | 1.00 | 1.00–1.00 | 0.664 | 1.00 | 1.00–1.00 | 0.815 | 1.00 | 1.00–1.00 | 0.785 | 1.00 | 1.00–1.00 | 0.570 |
|  | **Adj.** | 1.00 | 1.00–1.00 | 0.670 | 1.00 | 0.99–1.01 | 0.887 | 1.00 | 1.00–1.00 | 0.806 | 1.00 | 1.00–1.00 | 0.673 |
| **Malignancy** | **Unadj.** | 1.00 | 1.00–1.00 | 0.666 | 1.00 | 1.00–1.00 | 0.815 | 1.00 | 1.00–1.00 | 0.788 | 1.00 | 1.00–1.00 | 0.571 |
|  | **Adj.** | 1.00 | 1.00–1.00 | 0.671 | 1.00 | 1.00–1.01 | 0.886 | 1.00 | 1.00–1.00 | 0.807 | 1.00 | 1.00–1.00 | 0.674 |

Data are presented as unadjusted (upper row) and adjusted (lower row) OR, 95% CI and P value deriving from logistic regression analyses. Covariates in multivariable logistic regression models included age, sex, ancestry, Hispanic ethnicity, SLEDAI-2K and SDI scores at week 52 and the trial intervention. Statistically significant P values are in bold.

Adj., Adjusted; CI, confidence interval; MCS, mental component summary; OR, odds ratio; PCS, physical component summary; Unadj., unadjusted.
