# Supplementary material for: Adverse Health-Related Quality of Life Outcome Despite Adequate Clinical Response to Treatment in Systemic Lupus Erythematosus
Source: Front Med (Lausanne). 2021 Apr 16;8:651249. doi: 10.3389/fmed.2021.651249 (PMC8085308; doi:10.3389/fmed.2021.651249)
Supplement: Supplementary file 10 [file Table_10.DOCX]

**Supplementary Table 10.** Associations between SDI domains and adverse SF-36 mental subscales.

| **SDI domain** | | **VT** | | | **SF** | | | **RE** | | | **MH** | | |
| --- | --- | --- | --- | --- | --- | --- | --- | --- | --- | --- | --- | --- | --- |
|  |  | **OR** | **95% CI** | **P value** | **OR** | **95% CI** | **P value** | **OR** | **95% CI** | **P value** | **OR** | **95% CI** | **P value** |
| **Ocular** | **Unadj.** | 2.08 | 1.08–4.01 | **0.029** | 0.69 | 0.28–1.70 | 0.419 | 0.84 | 0.27–2.63 | 0.763 | 1.13 | 0.49–2.60 | 0.769 |
|  | **Adj.** | 1.49 | 0.75–2.96 | 0.257 | 0.50 | 0.20–1.27 | 0.146 | 0.61 | 0.19–1.99 | 0.414 | 0.81 | 0.34–1.93 | 0.629 |
| **Neuropsychiatric** | **Unadj.** | 1.00 | 0.56–1.76 | 0.990 | 1.55 | 1.01–2.38 | **0.044** | 1.23 | 0.66–2.29 | 0.517 | 1.59 | 1.00–2.53 | **0.050** |
|  | **Adj.** | 0.71 | 0.39–1.30 | 0.265 | 1.32 | 0.84–2.07 | 0.225 | 0.97 | 0.49–1.90 | 0.922 | 1.33 | 0.82–2.18 | 0.251 |
| **Renal** | **Unadj.** | 1.29 | 0.29–5.84 | 0.738 | 1.03 | 0.23–4.61 | 0.975 | 3.70 | 1.01–13.57 | **0.048** | 1.41 | 0.31–6.38 | 0.654 |
|  | **Adj.** | 1.50 | 0.30–7.51 | 0.624 | 0.96 | 0.20–4.48 | 0.953 | 3.21 | 0.80–12.85 | 0.100 | 1.21 | 0.25–5.83 | 0.811 |
| **Pulmonary** | **Unadj.** | 0.76 | 0.21–2.74 | 0.672 | 0.90 | 0.32–2.57 | 0.843 | 1.54 | 0.57–4.14 | 0.393 | 0.81 | 0.23–2.90 | 0.747 |
|  | **Adj.** | 0.70 | 0.18–2.72 | 0.604 | 0.77 | 0.26–2.33 | 0.648 | 1.17 | 0.38–3.59 | 0.785 | 0.73 | 0.19–2.74 | 0.637 |
| **Cardiovascular** | **Unadj.** | 1.57 | 0.67–3.67 | 0.296 | 1.04 | 0.42–2.59 | 0.937 | 2.11 | 0.84–5.25 | 0.111 | 1.10 | 0.40–3.00 | 0.856 |
|  | **Adj.** | 0.97 | 0.39–2.39 | 0.945 | 0.81 | 0.31–2.10 | 0.662 | 1.61 | 0.61–4.23 | 0.338 | 0.77 | 0.27–2.21 | 0.629 |
| **Peripheral vascular** | **Unadj.** | 1.48 | 0.75–2.92 | 0.262 | 0.60 | 0.20–1.77 | 0.353 | 0.42 | 0.06–2.80 | 0.369 | 0.54 | 0.14–2.04 | 0.362 |
|  | **Adj.** | 1.31 | 0.63–2.73 | 0.465 | 0.54 | 0.18–1.63 | 0.271 | 0.40 | 0.06–2.69 | 0.345 | 0.49 | 0.13–1.88 | 0.296 |
| **Gastrointestinal** | **Unadj.** | 1.52 | 0.64–3.64 | 0.346 | 0.55 | 0.17–1.80 | 0.327 | 1.62 | 0.57–4.57 | 0.362 | 0.49 | 0.12–2.04 | 0.329 |
|  | **Adj.** | 1.18 | 0.47–2.99 | 0.723 | 0.42 | 0.13–1.41 | 0.161 | 1.17 | 0.39–3.53 | 0.784 | 0.37 | 0.09–1.60 | 0.184 |
| **Musculoskeletal** | **Unadj.** | 1.37 | 0.93–2.01 | 0.114 | 1.08 | 0.72–1.62 | 0.724 | 0.75 | 0.37–1.52 | 0.418 | 0.74 | 0.41–1.33 | 0.313 |
|  | **Adj.** | 1.15 | 0.77–1.74 | 0.490 | 0.93 | 0.61–1.42 | 0.745 | 0.62 | 0.29–1.30 | 0.204 | 0.64 | 0.35–1.18 | 0.152 |
| **Skin** | **Unadj.** | 1.20 | 0.56–2.53 | 0.642 | 1.12 | 0.55–2.28 | 0.757 | 0.56 | 0.14–2.20 | 0.409 | 1.29 | 0.61–2.73 | 0.506 |
|  | **Adj.** | 0.97 | 0.43–2.17 | 0.942 | 0.97 | 0.46–2.03 | 0.936 | 0.45 | 0.11–1.81 | 0.259 | 1.11 | 0.50–2.44 | 0.806 |
| **Gonadal failure** | **Unadj.** | 1.00 | 1.00–1.00 | 0.793 | 1.00 | 1.00–1.00 | 0.782 | 1.00 | 1.00–1.00 | 0.821 | 1.00 | 1.00–1.00 | 0.798 |
|  | **Adj.** | 1.00 | 1.00–1.00 | 0.864 | 1.00 | 1.00–1.00 | 0.802 | 1.00 | 1.00–1.01 | 0.873 | 1.00 | 1.00–1.00 | 0.835 |
| **Diabetes** | **Unadj.** | 1.00 | 1.00–1.00 | 0.795 | 1.00 | 1.00–1.00 | 0.782 | 1.00 | 1.00–1.00 | 0.820 | 1.00 | 1.00–1.00 | 0.800 |
|  | **Adj.** | 1.00 | 1.00–1.01 | 0.866 | 1.00 | 1.00–1.00 | 0.802 | 1.00 | 1.00–1.00 | 0.872 | 1.00 | 1.00–1.00 | 0.836 |
| **Malignancy** | **Unadj.** | 1.00 | 1.00–1.00 | 0.796 | 1.00 | 1.00–1.00 | 0.785 | 1.00 | 1.00–1.00 | 0.822 | 1.00 | 1.00–1.00 | 0.800 |
|  | **Adj.** | 1.00 | 0.99–1.01 | 0.867 | 1.00 | 1.00–1.00 | 0.803 | 1.00 | 1.00–1.01 | 0.875 | 1.00 | 1.00–1.00 | 0.836 |

Data are presented as unadjusted (upper row) and adjusted (lower row) OR, 95% CI and P value deriving from logistic regression analyses. Covariates in multivariable logistic regression models included age, sex, ancestry, Hispanic ethnicity, SLEDAI-2K and SDI scores at week 52 and the trial intervention. Statistically significant P values are in bold.

Adj., Adjusted; CI, confidence interval; MCS, mental component summary; OR, odds ratio; PCS, physical component summary; Unadj., unadjusted.
